# Exploring Metastable Phases in Cerium-Doped Zirconia: Insights from X-ray Diffraction, Raman, X-ray Absorption, and Luminescence Spectroscopy

**DOI:** 10.1021/acs.inorgchem.5c00865

**Published:** 2025-05-08

**Authors:** Luiza B. F. dos Santos, Volodymyr Svitlyk, Selina Richter, Christoph Hennig, Katharina Müller, Elena F. Bazarkina, Kristina O. Kvashnina, Thorsten Stumpf, Nina Huittinen

**Affiliations:** † Institute of Resource Ecology, Helmholtz-Zentrum Dresden-Rossendorf, Bautzner Landstraße 400, 01328 Dresden, Germany; ‡ Institute of Chemistry and Biochemistry, Freie Universität Berlin, Fabeckstraße. 34-36, 14195 Berlin, Germany; § The Rossendorf Beamline (BM20), CS40220, European Synchrotron Radiation Facility, 38043 Grenoble Cedex 9, France

## Abstract

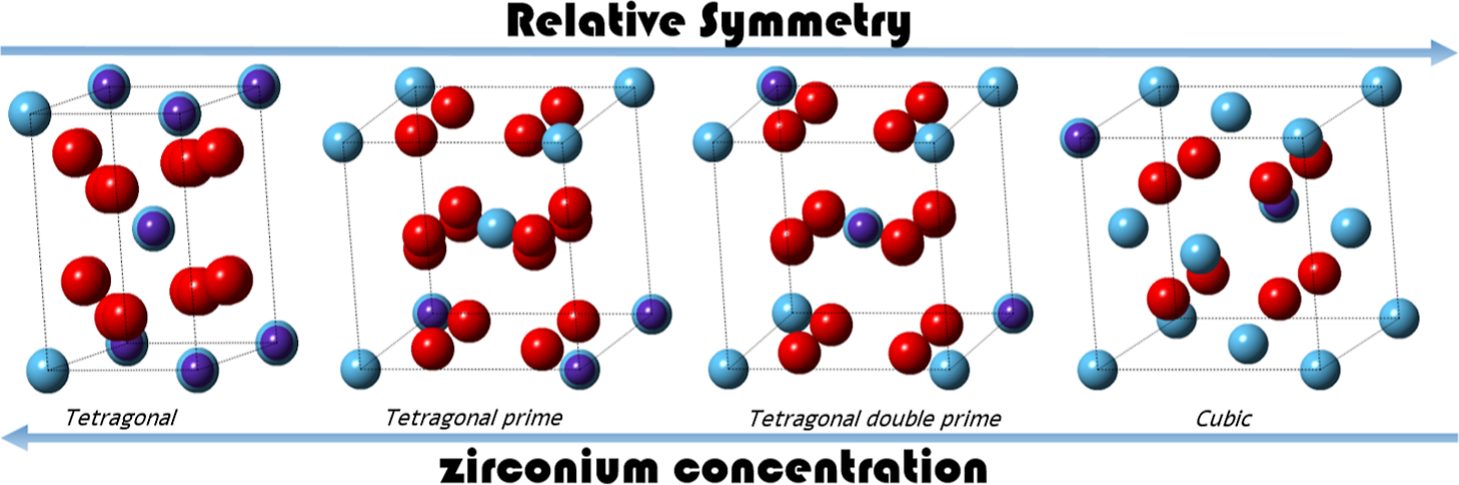

The ZrO_2_–CeO_2_ system is fundamental to various technological applications, yet unresolved questions persist regarding cation miscibility and the occurrence of metastable phases in the Zr_1–*x*_Ce_*x*_O_2_ phase diagram. This work addresses these gaps through a comprehensive investigation of Zr_1–*x*_Ce_*x*_O_2_ compositions with varying cerium concentrations and incorporating Eu^3+^ as a luminescent probe. Synchrotron powder X-ray diffraction analysis unveiled a miscibility gap between 20 and 50 mol % cerium. Beyond this gap, the formation of solid solutions and multiple crystalline phases was observed, including tetragonal prime (t′) and tetragonal double prime (t″) structures, depending on cerium content. Raman investigations revealed a unique distortion band in all compositions containing the t′ phase. Our high energy resolution fluorescence detected X-ray absorption near edge structure spectroscopy (HERFD-XANES) analysis implies that this feature results from oxygen ion displacement in the t′ structure. Luminescence spectroscopy of the europium environment revealed distinct excitation and emission spectra across the various crystal phases, enabling unambiguous identification of all metastable phases. These findings highlight the complex polymorphism of the ZrO_2_–CeO_2_ system. The ability to precisely control phase composition offers significant potential for optimizing properties for diverse applications, including oxygen sensors, three-way catalysts, and solid oxide fuel cells for clean, sustainable energy generation.

## Abstract 2

This study explores phase transformations in cerium-doped zirconia (ZrO_2_–CeO_2_) solid solutions. Synchrotron X-ray diffraction, Raman, and luminescence spectroscopy reveal tetragonal metastable phases and a miscibility gap between 20–50 mol % Ce. Raman and HERFD-XANES confirm that Ce remains tetravalent, with structural distortions arising from oxygen displacement rather than Ce^3+^ formation. These findings enhance our understanding of phase behavior, aiding the development of materials with improved oxygen storage and catalytic performance.

## Introduction

Lanthanide (Ln) and yttrium (Y) doped zirconia (ZrO_2_) solid solutions have gained significant attention in recent years owing to their versatile applications across multiple fields. These materials are used as oxygen sensors and in catalysis driven by their exceptional oxygen storage/release capacity (OSC), which is a critical property in technologies such as three-way catalysts.^1−4^ In addition, they are important for the development of solid oxide fuel cells (SOFCs), which have been highlighted as significant improvements toward the generation of sustainable and clean energy.^5−7^

The incorporation of Ln and Y into the zirconia matrix leads to modifications of the crystal structure. It undergoes several phase transformations, from the monoclinic (m) structure (space group *P*2_1_/*c*), which is the stable form of pristine ZrO_2_ at ambient pressure and temperature conditions, to tetragonal (t) (space group *P*4_2_/*nmc*) and cubic (c) (space group *Fm*3̅*m*) modifications, depending on several factors, such as the sintering temperature, the size of the metal cation, and its oxidation state. The stability range of metastable phases such as tetragonal prime (t′) and tetragonal double prime (t″) are still a matter of controversy. They have been reported to form in some specific Ln-stabilized ZrO_2_ compositions, but their formation is not completely understood, and their correct identification and distinction from tetragonal or cubic structures have proven difficult.^8−11^ These metastable phases exhibit deviations from the ideal tetragonal or cubic phases in their degree of tetragonality, characterized by the *c*/*a* ratio (*a* and *c* being lattice parameters), as well as slight variations in the oxygen *z* coordinates. Considering a normalized lattice parameter *a*_n_, which is obtained by multiplying the lattice parameter *a* by , the t′ phase is characterized by a *c*/*a*_n_ ratio between 1.01 and 1.001, i.e. significantly lower than 1.018 for the tetragonal structure.^12−14^ The t′ phase can be stabilized for example in yttrium-stabilized zirconia (YSZ) modifications via quenching, which avoids the formation of m + c-YSZ phase mixtures forming upon slow cooling.^15,16^ The t′ phases of YSZ are applied as thermal barrier coatings (TBCs) for gas turbine blades due to their outstanding mechanical properties.^17,18^ The t″ phase, especially encountered in some cerium (Ce)-stabilized zirconia compositions, is also referred to as a quasicubic structure. The multiple nomenclatures highlight the challenge of distinguishing this phase from the cubic structure, as it is frequently not identified as a metastable phase. The t″ phase has a *c*/*a*_n_ ratio of 1.00, i.e. the lattice parameters are identical to the cubic modification, however, the displacement of oxygen atoms from their crystallographic Wykoff positions 8c in a cubic lattice distinguishes the t″ phase from the true cubic ZrO_2_ structure. The displacement of oxygen anions results in vacancies at the crystallographic oxygen sites, which may result in enhanced oxygen storage capacities and improved electrical conductivities, making cerium-doped zirconia solid solutions promising candidates for advanced technological applications, especially in the fuel cell field.^1,4,6,19,20^ Understanding the structural evolution and phase transformations in these materials is thereby crucial for optimizing their performance and expanding their applicability.

Despite the considerable research related to Ce-doped zirconia materials, several fundamental questions regarding their structural features, phase behavior, and the impact of the different polymorphs on the resulting properties remain to be addressed.

Early studies of the ZrO_2_–CeO_2_ solid solution series have described the phase diagram solely with the help of m, t, and c solid solution compositions.^2,11,21,22^ Subsequent studies have introduced additional phase diagrams that incorporate metastable t′ and t″ phases, as well as binary compounds such as Ce_2_Zr_3_O_10_.^23^ However, the existing literature does not consistently align with the presence of various metastable phases that are not accounted for in the phase diagram established by Duwez and Odell.^12,21,24^ The synthesis conditions in these studies vary greatly, from quenched samples that have undergone annealing prior to investigations, to nanosized compounds, where additional phase stabilization effects can arise from the small size of the particles.^25^ Therefore, it is difficult to pinpoint the underlying reasons for the occurrence of the different phases in these studies and to assign specific synthesis conditions to the appearance of stable vs metastable phases in the Ce-doped zirconia or Zr-doped ceria compositions.

This work aims to increase the understanding of the formation of the different phases in the ZrO_2_–CeO_2_ solid solution series by investigating nonquenched bulk materials over the whole compositional range with a combination of analytical techniques capable of distinguishing the different phases in the samples. More specifically, high-resolution synchrotron powder X-ray diffraction (SPXRD) has been applied to identify the different structural polymorphs in the samples. For the specific identification of the t′ and t″ phases, Raman spectroscopy has been applied, due to the formation of a distortion band in Raman spectra. The Ce redox state has been investigated with high energy resolution fluorescence detected X-ray absorption near edge structure spectroscopy (HERFD-XANES). Finally, to explore local order–disorder phenomena in the samples, a small amount (0.1 mol %) of europium (Eu) was introduced to the solid solution compositions to allow for luminescence spectroscopic investigations. It is assumed that Eu^3+^ replaces Ce^4+^ in identical crystallographic positions due to its similar ionic radius.

By thoroughly grasping phase changes in Ce–ZrO_2_ matrices, this study aims to facilitate the understanding and subsequent predictions of their behavior, offering new opportunities for innovation across various fields, and driving advancements in materials science and technology.

## Results and Discussion

For a comprehensive investigation of phase transitions within cerium-doped zirconia systems, 25 compositions (Zr_1–*x*_Ce_*x*_O_2_) were synthesized via the coprecipitation route, with *x* ranging from 0.1 to 1.0. Additionally, 0.1 mol % of Eu^3+^ was introduced as a probe for luminescence analysis. Detailed synthesis procedures are described in the Experimental Section.

Synchrotron powder X-ray diffraction (SPXRD) analysis provides information about the phase composition and crystal structure in the samples. The high intensity of the synchrotron X-ray beam in combination with a high-resolution detector enables the identification of minor phases in the samples as well as the identification of the metastable tetragonal phases.^26^ The measured diffractograms of all sample compositions have been compiled in Figure S1. The results from Rietveld refinement have been summarized in Tables 1–3. A thorough discussion of the fitting parameters can be found in the Supporting Information, which is further substantiated by additional measurements compiled in Table S1 and Figure S2. The tetragonality ratios for all the compositions with a phase percentage higher than 5% and the scattering domain size, determined using the Debye–Scherrer equation, are shown in Table S2. The smallest crystallite size obtained in our Zr_1–*x*_Ce_*x*_O_2_ samples is 44 nm, which is larger than the critical crystallite size of 33 nm, below which additional dopant-independent stabilization of the crystal structure due to surface energy effects could take place.^25^ Consequently, the stabilization of the crystal structure can be attributed to dopant effects.

**Table 1:** Parameters Acquired from Rietveld Refinements of the Synchrotron X-ray Diffraction Data of Zr_1–*x*_Ce_*x*_O_2_ Compositions with 0.1 < *x* < 0.30

	0.10	0.11	0.12	0.13	0.14	0.15	0.16	0.17	0.18	0.22	0.26	0.30
***P*2_1_/*c* (m)**												
*a*_m_	5.19162(4)	5.1973(5)	5.20020(6)	5.20536(5)	5.20777(5)	5.21160(6)	5.21259(10)	5.2090(3)	5.21270(10)	5.19780(7)	5.15880(16)	
*b*_m_	5.22907(4)	5.2284(5)	5.22700(6)	5.22690(6)	5.22524(5)	5.22541(6)	5.22413(11)	5.2241(3)	5.21620(9)	5.23010(6)	5.27680(19)	
*c*_m_	5.36101(4)	5.3681(5)	5.37180(6)	5.38002(6)	5.38388(5)	5.38968(6)	5.39179(9)	5.3896(3)	5.40010(7)	5.40040(4)	5.37940(14)	
*x* (Zr)	0.273955	0.273543	0.27342	0.27322	0.27247	0.27242	0.27204	0.2731(6)	0.26004	0.24882	0.27404	
*y* (Zr)	0.038461	0.038212	0.03812	0.03777	0.03712	0.03697	0.03664	0.0353(5)	0.03819	0.02689	0.03161	
*z* (Zr)	0.209618	0.20969	0.20983	0.21037	0.21056	0.21050	0.21053	0.2127(5)	0.21329	0.22222	0.21283	
*x* (Ce)	0.273955	0.273543	0.27342	0.27322	0.27247	0.27242	0.27204	0.2731(6)	0.26004	0.24882	0.27404	
*y* (Ce)	0.038461	0.038212	0.03812	0.03777	0.03712	0.03697	0.03664	0.0353(5)	0.03819	0.02689	0.03161	
*z* (Ce)	0.209618	0.20969	0.20983	0.21037	0.21056	0.21050	0.21053	0.2127(5)	0.21329	0.22222	0.21283	
*x* (O1)	0.065113	0.065723	0.06468	0.06432	0.06130	0.06436	0.05323	0.019(4)	–0.00868	–0.03942	–0.00680	
*y* (O1)	0.325330	0.322980	0.32121	0.31984	0.31726	0.31378	0.30720	0.297(3)	0.27611	0.21395	0.24098	
*z* (O1)	0.353738	0.355613	0.35809	0.35907	0.36055	0.36274	0.36557	0.364(3)	0.34530	0.32018	0.28941	
*x* (O2)	0.451630	0.451950	0.45255	0.45398	0.45129	0.45356	0.46164	0.490(5)	0.47267	0.49246	0.50022	
*y* (O2)	0.755888	0.755421	0.75511	0.75462	0.75716	0.75766	0.75729	0.752(2)	0.75609	0.76804	0.76623	
*z* (O2)	0.477054	0.476260	0.4755	0.47533	0.47341	0.47261	0.47160	0.487(5)	0.52427	0.52221	0.35957	
Ce (occ)	0.10	0.11	0.12	0.13	0.14	0.15	0.16	0.17	0.18	0.22	0.26	
% phase	97.9(6)	96.4(6)	94.0(5)	91.7(6)	71.2(3)	47.6(2)	16.5(1)	6.7(1)	2.1(3)	4.3(4)	1.1(1)	
***P*4_2_/*nmc* (t)**												
*a*_t_	3.62794(9)	3.6299(3)	3.63010 (4)	3.63208(9)	3.63177(2)	3.63246(15)	3.63260(8)	3.6346	3.63706(10)	3.64603(6)	3.64600(2)	3.64571(7)
*c*_t_	5.2262 (3)	5.2288(7)	5.22960(7)	5.23141(19)	5.22828(5)	5.22839(3)	5.22898(15)	5.231	5.23478(17)	5.24385(11)	5.24450(3)	5.24435(13)
*z* (O)	0.24(4)	0.224(8)	0.22000(3)	0.21400(2)	0.20540(6)	0.20440(3)	0.20417(16)	0.20436	0.20408	0.20586	0.20575	0.20486
Ce occ	0.10	0.11	0.12	0.13	0.14	0.15	0.16	0.17	0.18	0.22	0.26	0.30
% phase	2.1(1)	3.7(1)	5.6(6)	8.3(1)	28.8(8)	52.4(2)	83.5(3)	93.3(3)	97.0(1)	91.2(1)	81.8(3)	66.1(1)
***P*4_2_/*nmc* (t′ and t″)**												
*a*_t_									3.72496(8)	3.72604 (4)	3.72740(2)	3.72552(2)
*c*_t_									5.30570(18)	5.30803(9)	5.31230(3)	5.31142(4)
*z* (O)									0.42300(6)	0.46400(2)	0.46990(9)	0.46813
Ce (occ)									0.18	0.22	0.26	0.30
% phase									0.89(1)	4.45(2)	17.08(4)	33.8(1)
*R*_p_	6.67	7.59	8.04	7.78	5.78	5.20	4.02	4.01	3.59	4.71	5.15	3.67
*R*_wp_	9.13	10.16	10.77	10.47	8.01	7.32	5.68	5.61	5.48	3.18	3.44	5.27
*s*	2.6893	2.9158	3.0093	2.9127	2.2808	2.0683	1.5720	1.5685	1.4913	1.2111	1.3553	1.2737

**Table 2:** Parameters Acquired from Rietveld Refinements of the Synchrotron X-ray Diffraction Data of Zr_1–*x*_Ce_*x*_O_2_ with 0.42 < *x* < 0.70

	0.42	0.50	0.58	0.60	0.65	0.70	0.70^a^
***P*4_2_/*nmc* (t)**							
*a*_t_	3.64410(2)	3.64340(2)	3.64674	3.64597	3.65404(12)	3.86700(2)	
*c*_t_	5.24400(3)	5.24435(3)	5.24847	5.24704	5.25830(3)	4.71800(4)	
*z* (O)	0.20471	0.20687	0.20492	0.21204	0.207(8)	0.192442	
Ce occ	0.42	0.50	0.58	0.60	0.65	0.70	
% phase	37.2(1)	18.9(1)	7.2(1)	3.7(1)	1.1(1)	3.7(1)	
***P*4_2_/*nmc* (t′ and *t*″)**							
*a*_t_	3.72400(2)	3.72590(2)	3.73443(5)	3.75034(4)	3.75716(8)	3.76540(8)	
*c*_t_	5.30990(3)	5.31140(3)	5.31707(8)	5.31959(9)	5.33145(9)	5.32500(8)	
*z* (O)	0.46960(3)	0.47080(3)	0.47360(9)	0.47020(6)	0.47438	0.48490	
Ce (occ)	0.42	0.50	0.58	0.60	0.65	0.70	
% phase	62.8(1)	81.1(2)	92.8(3)	96.3(4)	98.9(4)	96.3	
***Fm*3̅*m* (c)**							
*a*_c_							5.32926(13)
*z* (O)							0.25
Ce (occ)							0.7
% phase							100.0(2)
*R*_p_ (%)	3.62	3.42	8.18	8.9	8.75	5.52	6.48
*R*_wp_ (%)	5.41	5.16	13.04	13.79	13.61	8.74	10.45
*s*	1.1437	1.0735	2.5953	1.9017	2.0463	1.0158	1.2150

a Additional refinement considering the phase purely cubic.

**Table 3:** Parameters Acquired from Rietveld Refinements of the Synchrotron X-ray Diffraction Data of Zr_1–*x*_Ce_*x*_O_2_ with 0.75 < *x* < 1.0

	0.75	0.75^a^	0.80	0.80^a^	0.85	0.90	1.00
***P*4_2_/*nmc* (t′ and *t*″)**							
*a*_t_	3.76927(5)		3.77345(2)				
*c*_t_	5.32411(15)		5.33182(5)				
*z* (O)	0.487053		0.489622				
Ce (occ)	0.75		0.80				
% phase	100.0(2)		100.0(3)				
***Fm*3̅*m* (c)**							
*a*_c_		5.32635(17)		5.35180	5.36900(3)	5.39240(2)	5.411021(5)
*z* (O)		0.25		0.25	0.25	0.25	0.25
Ce (occ)		0.75		0.80	0.85	0.90	1.00
% phase		100.0(3)		100.0(3)	100.0(6)	100.0(7)	100.0(3)
*R*_p_ (%)	5.79	5.35	5.45	5.62	6.1	5.54	5.54
*R*_wp_ (%)	7.89	8.03	7.24	7.33	8.66	8.69	8.13
*s*	1.0391	1.0575	1.0017	1.0130	1.0543	1.0974	1.0272

a Additional refinement considering the phase purely cubic.

**Figure 1:**
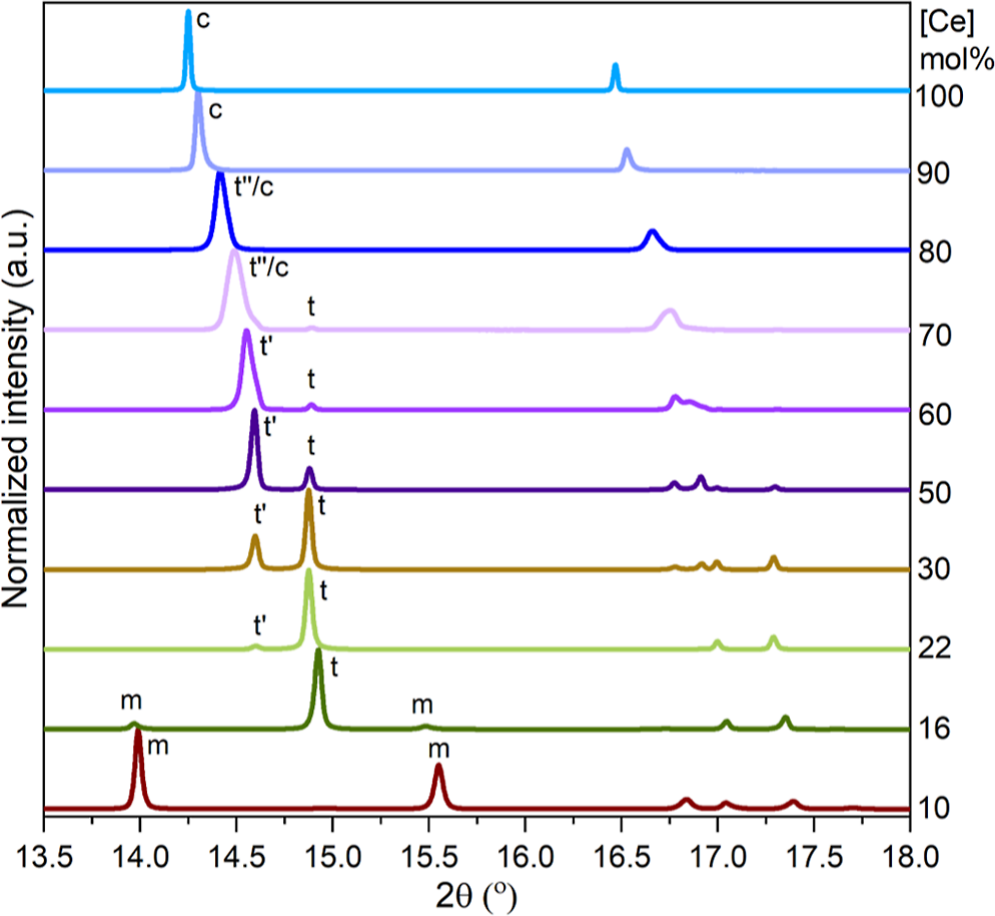
SPXRD diffractograms (λ = 0.774901 Å) of Zr_1–*x*_Ce_*x*_O_2_ (0.1 ≤ *x* ≤ 1.0) with different crystal phases, monoclinic (m), tetragonal (t), tetragonal prime (t′), tetragonal double prime (t″), and cubic (c).

**Figure 2:**
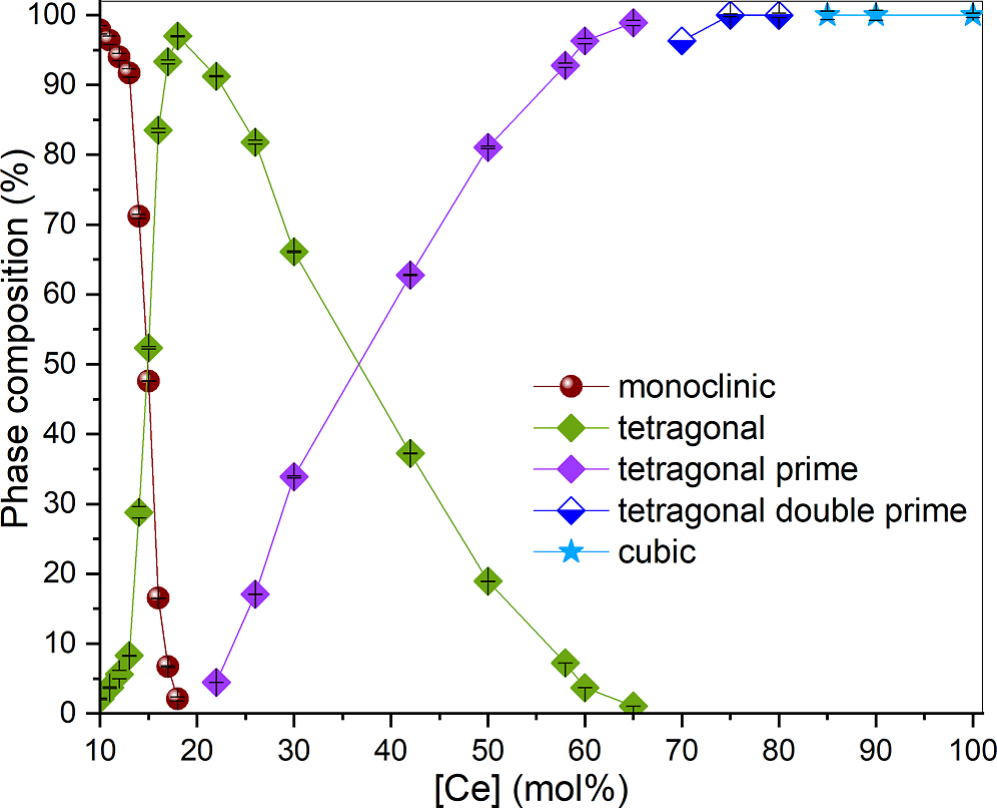
Quantitative distribution of Zr_1–*x*_Ce_*x*_O_2_ phases obtained from the Rietveld refinement. In some cases, the error bars are smaller than the symbols.

Figure 1 shows a representative section of the diffractograms with the strongest reflections of the m-, t- and c-Zr_1–*x*_Ce_*x*_O_2_ phases. In agreement with the literature, the compounds with low cerium doping, reveal the monoclinic (m) phase by two characteristic reflections (111̅) and (111) at 2θ = 13.99 and 15.53°, respectively.^27^ The monoclinic phase predominates when the cerium concentration is below 15 mol %. The quantitative data refinement (Table 1) shows that the compound with 15 mol % Ce exhibits approximately equal percentages of monoclinic and tetragonal phases, as shown in Figure 2.

With increasing cerium concentration, the tetragonal t-Zr_1–*x*_Ce_*x*_O_2_, characterized by its (101) reflection at 2θ = 14.95°, becomes predominant (indicated in Figure 1 with t). For Ce concentrations ranging from 17 to 26 mol %, three crystalline phases are observed, however, some exhibit concentrations less than 5% (Table 1). Given the potential for fitting errors of weak features in Rietveld refinement, these phases were not considered in the discussion.^28,29^

In the samples containing 16–30 mol % Ce, the predominant phase is tetragonal (t). The samples show *c*/*a* ratios (Table S2) of around 1.43, which are in agreement with the literature data.^12^ A third phase becomes visible in samples with more than 18 mol % Ce, with a Bragg reflection at 14.73° (indicated with t′ in Figure 1). As the doping concentration increases, the characteristic peak for this phase becomes more pronounced and broader. To determine whether or not this reflection is related to a tetragonal metastable phase, the strategy used by e.g., Varez et al.,^10^ was employed. This method involves normalizing the unit cell parameters by multiplying the lattice parameter *a* by  as shown in eq 1. This normalization highlights the relationship between the cubic and pseudocubic structures,^30^ represented by the normalized lattice parameter *a*_n_
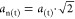
1

**Figure 3:**
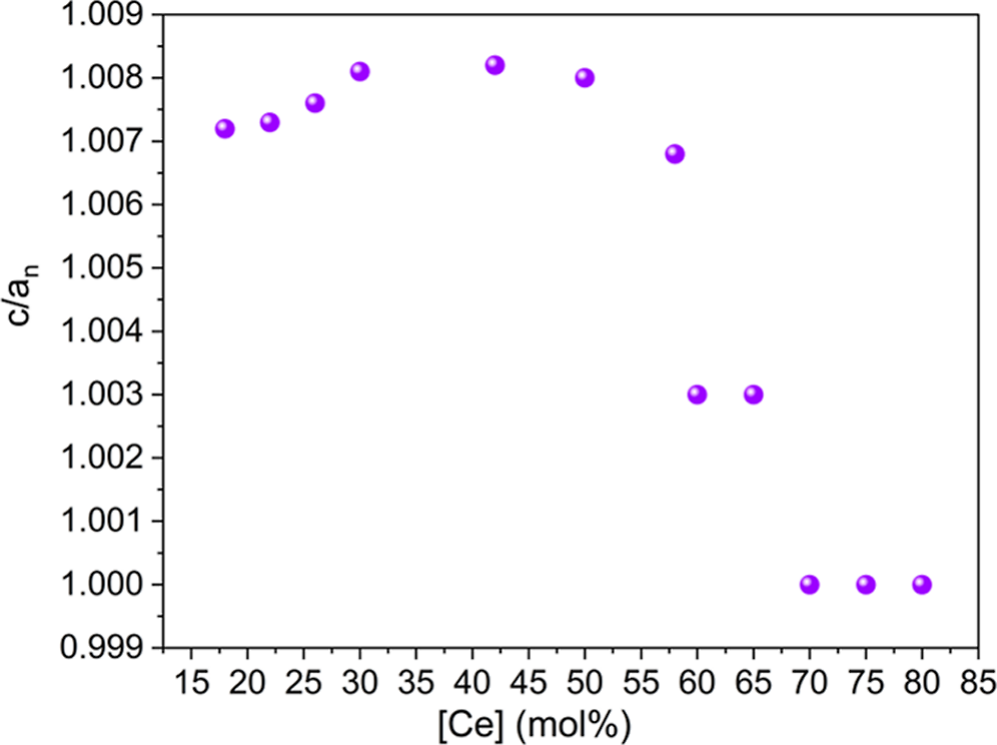
Tetragonality determined by *c*/*a*_n_ (normalized *a*), for a tetragonal prime and a tetragonal double prime structure in Zr_1–*x*_Ce_*x*_O_2_ (0.18 ≤ *x* ≤ 0.80).

To confirm the presence of t′ or t″, *c* divided by the normalized *a* (*a*_n(t)_) should approach 1.018 for the t phase, should be 1.001–1.018 for the t′ phase, and should equal 1.00 for the t″ phase, similar to what is expected for a pure cubic structure (*c*/*a* = 1).^10,12^ The *c*/*a*_n(t)_ ratios shown in Figure 3 suggest that the observed new phase is related to t′ in the composition range between 22 and 65 mol % Ce. The *c*/*a*_n(t)_ ratio for this phase is almost constant at 1.007–1.008 until 58 mol % Ce, thereafter the ratio decreases to 1.003 at 60 and 65 mol % Ce. At 70 mol %, the ratio further drops to 1.00, which is the first indication for potential t″ phase formation. Discussions in the literature make it questionable whether it is possible to distinguish the t″ from the cubic phase solely based on XRD.^8,10^ Nevertheless, we performed Rietveld refinement using synchrotron data. In the composition range from 70 to 100 mol % Ce, the data were fitted with both the metastable tetragonal and the cubic atom coordinates. The refinement of samples containing 70–80 mol % Ce yielded very similar *z* coordinates for the oxygen atoms (Figure S3) for the t″-Zr_1–*x*_Ce_*x*_O_2_ phases, which are around 0.47 and 0.49, respectively. Attempting to fit the data using fixed atomic parameters of the cubic structure resulted in a larger *R*-value, indicating this approach is unsatisfactory and strongly suggestive of the presence of the t″ phase. The coordinates for metastable phases are clearly larger than in the t-Zr_1–*x*_Ce_*x*_O_2_ and c-Zr_1–*x*_Ce_*x*_O_2_ phases with *z*(O) = 0.22 and 0.25, respectively, which serves as additional evidence for the presence of the t′ and/or t″ phase in the samples. The large values for the oxygen *z*(O) coordinate for both of the metastable phases can be understood as a displacement of oxygen atoms from their crystallographic Wyckoff position 8c. Above 80 mol % Ce, the small reflection of the tetragonal phase at 2θ = 14.89° disappears, and at 90 mol % Ce, a purely cubic phase (c) evolves with the peak (111) around 2θ = 14.4° (Figure S4). Due to the superior fit of the cubic phase for the 90 and 100 mol % Ce, the refinement involving the t″ phase will be not considered.

**Figure 4:**
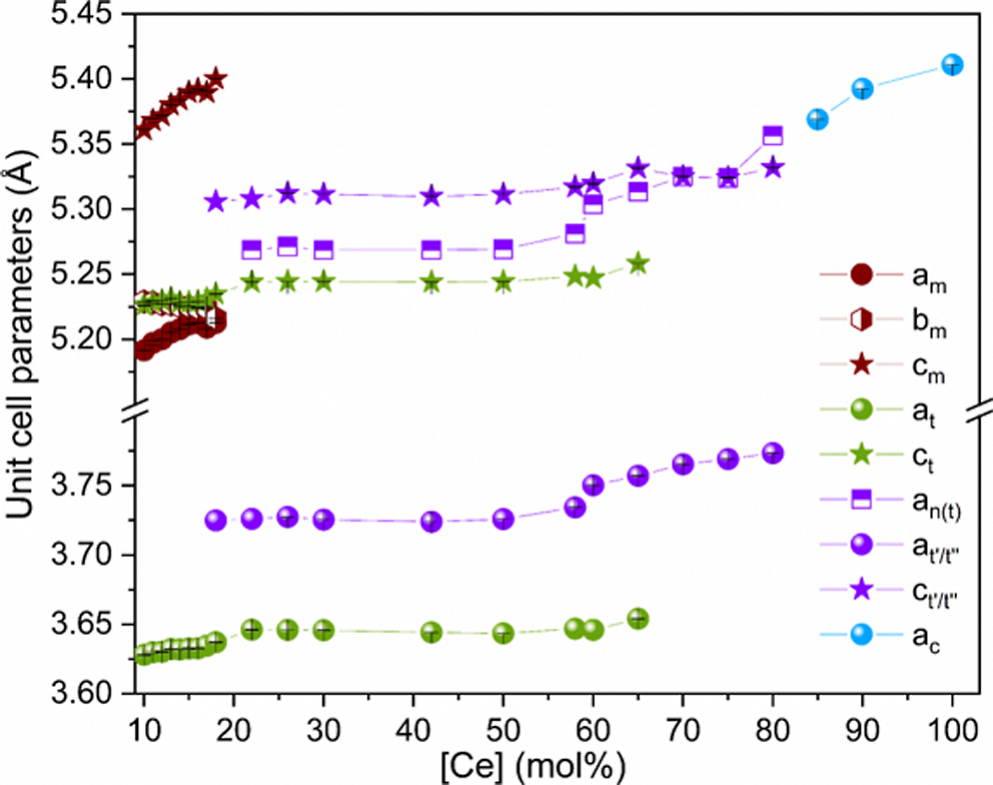
Lattice parameters of Zr_1–*x*_Ce_*x*_O_2_ (0.1 ≤ *x* ≤ 1.0), with *a*, *b*, and *c* for the monoclinic (m); *a* and *c* for the tetragonal (t); *a*, *a*_n(t)_ and *c* for tetragonal metastable (t′/t″); and *a* for the cubic (c) phase. The error bars are smaller than the data points.

The lattice parameters (Figure 4) for the monoclinic phase follow Vergard’s law as a function of chemical composition and display a nearly linear behavior. With increases of the Ce concentration, the *a* parameter of m-Zr_1–*x*_Ce_*x*_O_2_ (*a*_m_) increases from 5.1919 to 5.2126 Å, while *b*_m_ decreases from 5.2294 to 5.2224 Å. The lattice parameters *a*_m_ and *b*_m_ finally approached the same value, indicating a transformation from the monoclinic to the tetragonal phase. In addition, from 15 to 18 mol % Ce, an expansion of the tetragonal lattice caused by the incorporation of the larger Ce cations is visible, which is typical for homogeneous solid solutions. This behavior changes when the Ce concentration increases from 22 to 50 mol % Ce. The lattice parameters remain practically constant with *c*_t_ around 5.2438 Å at 22 mol % Ce, and 5.2443 Å at 50 mol % Ce. Similar behavior is observed for the *a*_n(t)_ and *c*_t′/t″_ parameters. Figure S4d, shows that the only difference between 22 and 50 mol Ce % is the intensity of the diffraction peaks, which decreases for the primary tetragonal phase, while it increases as a function of cerium concentration for the metastable tetragonal phase. For better visualization of the Bragg reflection positions in this compositional range, the reflections have been normalized to a maximum intensity of 1 in Figure S4b–d. The constant lattice parameters suggest a miscibility gap in this concentration range with two coexisting stoichiometric phases. At lower Ce concentrations, the systematic shift of the Bragg reflection at 14.59° to lower 2θ values is interrupted at 22 mol % Ce, indicating that the solubility limit of cerium related to the primary tetragonal phase has been reached. Beyond 22 mol %, a slight variation of the Bragg reflection position can be seen, suggesting a slightly different Ce content in the solid phase. Taking into account the quantitative phase content of the monoclinic, tetragonal, and tetragonal prime phases for samples with Ce concentrations between 18 and 50 mol %, as well as the overall Ce-doping, an approximate stoichiometry for the solubility limiting solid phase for Zr-rich compositions of 20 ± 2 mol % Ce was calculated. Hence, one of the stoichiometric phases can be identified as Zr_0.80_Ce_0.20_O_2_ (Zr_4_CeO_10_) with a tetragonal structure. This is a slightly higher Ce concentration than reported in Tani et al. and Yashima et al.,^31,32^ where the solubility limit of cerium was shown to depend on the synthesis methodology and the sintering temperature. In these studies, a maximum solubility of 16–18 mol % Ce in t-zirconia was found for sintering temperatures below 1500 °C.^31−34^ For Ce concentrations above 22 mol % Ce in the ZrO_2_ matrix, a second stoichiometric phase must incorporate the remaining cerium. By examining the systematic shift of the Bragg reflection at 14.59° (t′), a constant 2θ value is obtained at 50 mol % Ce, above which it shifts to lower 2θ values. Assuming the earlier calculated Zr_0.80_Ce_0.20_O_2_ stoichiometry of the Zr-rich phase and taking into account the phase percentages and the overall Ce concentration in the samples with 18–58 mol % Ce, a Ce solubility of 52 ± 6 mol % could be calculated. This stoichiometric phase is very likely Zr_0.5_Ce_0.5_O_2_, with a tetragonal prime structure. The existence of a stoichiometric Zr_0.5_Ce_0.5_O_2_, solid phase (ZrCeO_4_) has been reported in several studies due to its specific interest in the oxygen storage/release capacity (OSC) application.^35^ This crystal phase has been described as tetragonal with higher anharmonicity of the Zr–O_2_ subshell, as quasicubic (t″), cubic, or even as a mix of tetragonal and cubic phases.^9,10,35−37^

Beyond 50 mol % Ce, the Bragg reflection at 14.56° (t′ peak (101)) shifts further to lower 2θ values (Figure S4d), and the lattice parameters *a*_n_ and *c*_t′/t″_ increase progressively until they reach the value of *a*_c_ in the 70 mol % Ce composition (Figure 4). From there onward, all (identical) lattice parameters continue to increase linearly in accordance with Vegard’s law until the maximum value of 5.4110 Å for cerium dioxide is reached. This value is very close to 5.4123(1) Å reported for CeO_2_ powders synthesized by precipitation, hydrothermal, or sol–gel synthesis.^8,38^ It is noteworthy that between 70 and 80 mol % Ce, the lattice parameters corresponding to the tetragonal metastable and cubic phases are nearly identical, independent of the phase used for the refinement.

In summary, our SPXRD results showed the presence of m-Zr_1–*x*_Ce_*x*_O_2_ in compositions rich in zirconium (<16 mol % Ce). From 16 mol % Ce, a tetragonal Zr_1–*x*_Ce_*x*_O_2_ solid solution is formed with a solubility limit of 20 mol % Ce, resulting in the formation of a stoichiometric Zr_0.80_Ce_0.20_O_2_ (Zr_4_CeO_10_) solid phase with tetragonal structure. Between 20 and 50 mol % Ce, a miscibility gap exists. The solubility limit of Zr in Ce-rich compositions is 50%, leading to the formation of the stoichiometric ZrCeO_4_ phase with a t′ structure. At compositions with higher Ce than Zr concentrations, a metastable t′ solid solution transforms to metastable t″, and finally to c-Zr_1–*x*_Ce_*x*_O_2_ and c-CeO_2_.

### Raman and HERFD-XANES

**Figure 5:**
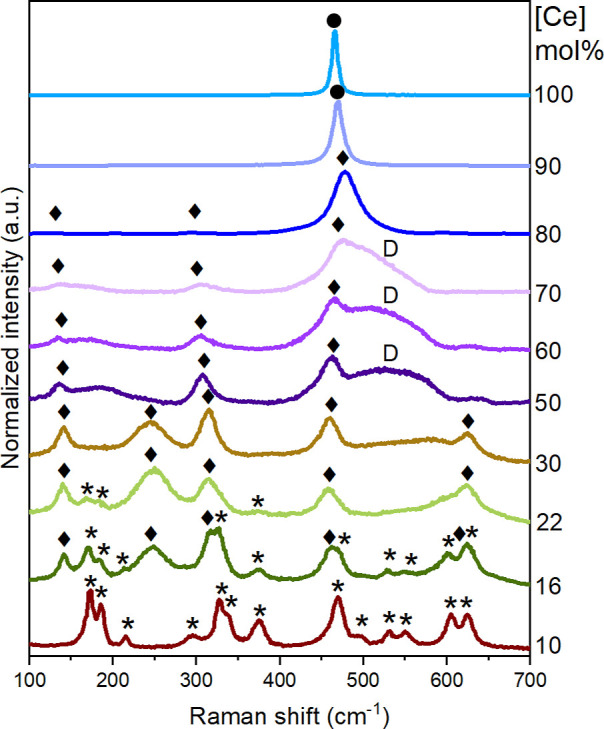
Raman spectra of Zr_1–*x*_Ce_*x*_O_2_ (0.1 ≤ *x* ≤ 1.0) with assigned bands representing the monoclinic (*), tetragonal (⧫), and cubic (●) phases. In addition, D is related to the distortion band.

Raman spectroscopy is a sensitive technique that can provide valuable insights into compositions, crystal phases (including metastable ones), order–disorder, and plastic deformations in crystalline samples.^39^ This technique is considered crucial for determining high compressive stress and identifying oxygen defects/vacancies in metal oxides.^40,41^ Thus, Raman spectroscopy should be able to confirm the presence of additional metastable phases, such as the t″ phase, which could not be unambiguously determined from our SPXRD data. Raman spectra were collected at ambient temperature for all Ce–ZrO_2_ compositions (Figures S5 and S6). Corresponding band assignments and vibrational modes are given in Table 4. Selected Raman spectra are presented in Figure 5.

**Table 4:** Frequencies (cm^–1^) and Symmetry Assignments of the Raman Active Vibrational Modes for the Cerium-Doped Zirconia Compounds: Monoclinic (*), Tetragonal (⧫), and Cubic (●) Phases

[Ce] (mol %)	1 (⧫) E_g_	2 (*) A_g_	3 (*) B_g_	4 (*) A_g_	5 (⧫) E_g_	6 (*) A_g_	7 (⧫) B_1g_	8 (*) B_g_	9 (*) A_g_	10 (*) B_g_
10		173.53	185.71	215.32		296.57		328.35	337.85	375.4
11		172.68	185.63	215.32		293.57		328.35	337.70	374.74
12		172.48	185.22	215.32		292.72		328.35	337.70	374.74
13		172.49	184.74	214.48		291.97		328.35	335.79	374.58
14		171.26	184.24	213.99		288.18		326.54		373.27
15		171.26	184.24	213.75		287.20		326.54		373.92
16	142.43	170.90	183.33	213.28	249.75		316.71	326.54		373.92
17	141.93				249.75		316.71			
18	141.77				249.75		316.71			
22	140.89	170.90	183.30		249.75		314.93			
26	140.89				247.98		314.93			
30	140.89				247.98		314.93			
42	140.89				247.98		314.93			
50	137.17						309.58			
58	136.68						305.29			
60	136.52						305.29			
65	136.52						304.03			
70	136.52						304.03			
75	135.47						302.51			
80	135.47						299.89			
85										
90										
100										

[Ce] (mol %)	11 (⧫) E_g_	12 (*) A_g_	13 (●) F_2g_	14 (*) B_g_	15 (*) B_g_	16 (*) A_g_	DB	17 (*) B_g_	18 (*) A_g_	19 (⧫) A_1g_
10		470.33		499.58	531.64	552.14		604.93	625.42	
11		470.33		499.58	529.84	552.14		604.93	625.42	
12		470.33		498.02	530.66	552.14		604.93	625.42	
13		470.33		496.78	530.66	551.16		604.93	622.63	
14		468.53		496.78	528.86	550.17		601.33	623.62	
15		468.53		496.78	528.86	550.17		601.33	623.62	
16	461.14	468.53			528.86	550.17		601.33	623.62	
17	460.16									628.83
18	460.16									628.83
22	459.81						603.20			626.04
26	459.81						602.87			626.04
30	459.81						593.57			626.04
42	459.81						564.02			
50	462.53						536.12			
58	462.53						525.44			
60	464.14						516.32			
65	464.01						516.49			
70	472.37						513.62			
75	474.17						511.01			
80	477.78									
85			475.40							
90			469.85							
100			465.56							

As already confirmed with SPXRD analysis, at low doping concentrations (≤22 mol % Ce), the Raman spectra exhibit characteristic vibrational bands for the monoclinic and tetragonal phases, with the monoclinic phase being predominant for zirconate compositions with cerium concentrations up to 15 mol %. Theoretically, the monoclinic ZrO_2_ structure displays 18 Raman A_g_ and B_g_ vibrational modes. Polarization effects limit the identification of vibrational modes to only 13, denoted with an asterisk (*) in Figure 5.^42,43^ These Raman bands exhibit a small shift to lower frequencies, suggesting bond lengthening due to the substitution of Zr cations by heavier Ce cations.^44^ For the tetragonal phase, 18 phonon branches are predicted, encompassing 15 optical vibration modes divided into 9 fundamental Raman active modes.^45^ Above 16 mol % Ce, the tetragonal phase predominates and exhibits five of these Raman active vibrational modes indicated with diamonds (⧫) in Figure 5.

Consistent with the SPXRD data, when increasing the doping concentration to 22 mol % Ce, all the five modes (3E_g_ + 1B_g_ + A_1g_) are shifted to lower frequencies as a result of Ce incorporation (Table 4).^43,44,46^

For concentrations above 22 mol % Ce, a broad band appears in the Raman spectra at approximately 600 cm^–1^. It increases in intensity and successively shifts to around 515 cm^–1^ as a function of increasing Ce concentration, and becomes clearly visible between 42 and 70 mol % Ce, which is the range where the tetragonal metastable phase(s) are predominant according to SPXRD data. In the literature, this broadband is associated with disorder or defect structures, and it is commonly reported as the D-band, indicated with the letter D in Figure 5.

The Raman spectra of the compositions with 22–42 mol % Ce differ only concerning the presence of this D-band, while the positions of all other five modes remain almost constant (Table 4). This provides additional evidence of the presence of stoichiometric Ce and Zr containing phases and, consequently a miscibility gap within this compositional range.

Different reasons for the appearance of the D-band have been postulated, and in some studies, the split into two individual bands has been indicated, referred to as D1, and D2, respectively.^47^ These D-bands have been assigned to two different coordination geometries, where a trivalent cation is coordinating with one or two oxygen vacancies V_O_^••^ (D1) or where the trivalent cation resides in a MO_8_-type environment without direct coordination with a vacancy (D2). The presence of a trivalent cation and the subsequent presence of the D-band in our Raman spectra could either be attributed to the presence of 1000 ppm Eu^3+^, added as a luminescent probe to the samples, or due to Ce^4+^ reduction to Ce^3+^, with the subsequent formation of oxygen vacancies, according to eq 2.^48^
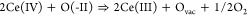
2

**Figure 6:**
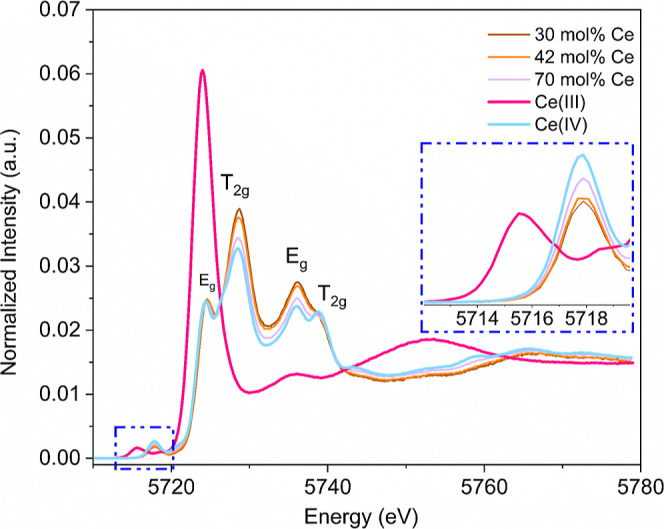
Ce L_III_-edge HERFD-XANES spectra with magnification of the Ce L_III_-pre-edge of 30, 42, and 70 mol % cerium-doped zirconia compositions, as well as CeO_2_ and CePO_4_ spectra as references for Ce(IV) and Ce(III), respectively.

The potential influence of Eu^3+^ on the formation of the D-band was investigated for some identically prepared samples, synthesized without Eu^3+^. Figure S7 shows that the appearance of the D-band is independent of the presence of europium. Thereby, the observed high concentration of defects may be attributed to an additional factor, as a consequence of potential Ce reduction. Thus, the cerium valence state in the samples was investigated with the XANES method in HERFD mode using an X-ray emission spectrometer.^47,49^ The analysis was performed on three selected samples with concentrations of 30, 42, and 70 mol % Ce in the samples. Corresponding Raman spectra showing the clear presence of the D-band in these samples have been compiled in Figure S8. CePO_4_ and CeO_2_ were used as redox reference standards for Ce(III) and Ce(IV), respectively. The Ce HERFD-XANES spectra at the L_III_ edge are shown in Figure 6. The peaks correspond to dipole-allowed electron transitions from the 2p_3/2_ to 5d_5/2_ with the preedge peak appearing around 5718.3 eV, originating from the 2p transition to a mixed 5d–4f valence state.^48,50^ The spectra for synthesized compounds closely match the reference spectrum for Ce(IV), showing two E_g_ bands at 5723.9 and 5736.0 eV, and two T_2g_ bands at 5728.5 and 5738.6 eV.^51^ This assignment, combined with the absence of the prepeak typically associated with Ce(III), indicates that all the cerium ions exhibit the 4+ oxidation state, contradicting the hypothesis proposed by many authors regarding the presence of Ce(III).^13,48^ The presence of the D-band, but the absence of trivalent cations, suggests a defect that is not associated with oxygen vacancies formed due to charge compensation in the lattice. Such a defect could be the displacement of some oxygen ions from their crystallographic positions to different z-coordinates, as observed in our SPXRD investigations (Figure S3).

The oxygen displacement and the clear presence of the D-band can be observed in Ce-doped ZrO_2_ compositions, where the presence of the metastable phase was confirmed (Figure 2). The Raman spectra were thereby carefully analyzed to confirm the presence of the t″ phase as indicated by SPXRD, and the Lorentz mathematical model was used to decompose the Raman bands for compositions between 65 and 90 mol % Ce. Figure S9 provides a detailed representation of the observed bands. Importantly, this analysis demonstrates that the D-band remains present as a peak at approximately 513 cm^–1^ up to 65 mol % Ce. At higher Ce concentrations, the D-band intensity decreases and its contribution is absorbed into a large band at around 475 cm^–1^. This band persists until 75 mol % Ce, after which it disappears entirely at 80 mol % Ce. Besides the presence of the D-band in the compositions between 22 and 75 mol % Ce, Raman bands at around 136, 305, and 470 cm^–1^ can be discerned. These three vibrational modes are also present in the sample with 80 mol % Ce, and they are clearly not related to the cubic phase, but rather to tetragonal-like phases. This confirms that the samples containing 70–80 mol % Ce indeed contain a tetragonal metastable phase. With increasing cerium content above 80 mol %, only the F_2g_ vibrational mode is present, and the D-band has fully disappeared. The F_2g_ band becomes successively narrower (Figure S10), indicating the transformation to a defect-free cubic structure (marked with ● in Figure 5).

Additionally, there is a notable shift to lower frequencies from 478 cm^–1^ for 80 mol % Ce to 466 cm^–1^ for CeO_2_, suggesting the stretching of chemical bonds.^44^ In a cubic structure, only one F_2g_ mode is Raman-active; this is the case for 90 mol % Ce. For the pure CeO_2,_ the spectrum agrees with the fluorite type found in literature, with the F_2g_ mode at around 465 cm^–1^.^44^

In summary, the Raman technique was effective in distinguishing the metastable t′ and t″ phases via the presence of the D-band and Raman vibrational modes belonging to the tetragonal structure. The cubic phase only forms above 80 mol % Ce, confirming our assumptions in the SPXRD investigations. We could also show, using the HERFD-XANES method, that Ce reduction does not take place in the samples, and the presence of the D-band can thereby only be attributed to the oxygen ion displacement in the metastable structures in comparison to the ideal tetragonal and cubic ones.

### Luminescence Spectroscopy

**Figure 7:**
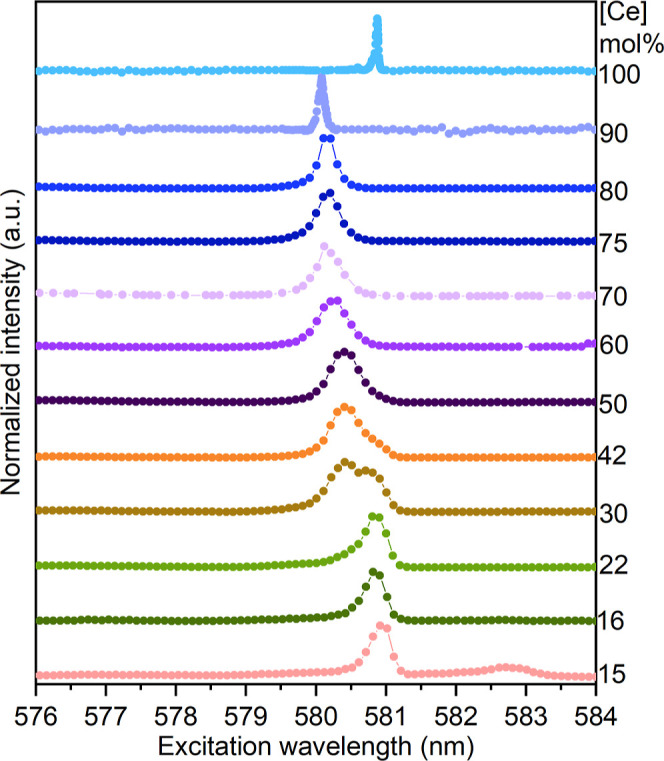
Eu^3+^ excitation spectra for synthesized Zr_1–*x*_Ce_*x*_O_2_ (0.15 ≤ *x* ≤ 1.0) compositions.

As a fourth, somewhat explorative method to investigate phase transformations in Ce-doped ZrO_2_, the excellent luminescent properties of the trivalent Eu^3+^ ion were utilized in luminescence spectroscopic investigations. Although the oxidation state of the host and luminescent cations do not match, Eu^3+^ luminescence is sensitive to local order–disorder phenomena, which will strongly depend on the phase composition of the host phase, as well as on other structural changes such as the dislocation of oxygen anions from their crystallographic positions.^52−56^ Selective excitation of the Eu^3+^ ion from the ^7^F_0_ ground state to the ^5^D_0_ excited state, both of which are nondegenerate, allows for the determination of the number of nonequivalent environments present in the solid matrix. To gain a deeper understanding of the local environment surrounding the Eu^3+^ cations within cerium-doped zirconia compositions, experiments using site-selective laser excitation were conducted at a low temperature (below 10 K) for selected Ce concentrations.^27^Figure 7, displays excitation spectra (integrated luminescence intensity as a function of excitation energy) of the ^5^D_0_ → ^7^F_0_ transition in these Ce-doped ZrO_2_ compositions. Nonstacked spectra can be found in the Supporting Information (Figure S12). The signals in Figure 7 correspond to the different structural Eu^3+^ environments in the samples. The position and intensity of each signal provide initial indications of the respective Eu^3+^ environments, where a stronger ligand field generally leads to a lower energy transition and a stronger bathochromic shift of the signal.^26,52^ To better understand the phase transformation, the excitation spectrum is examined from the higher to lower wavelengths.

**Figure 8:**
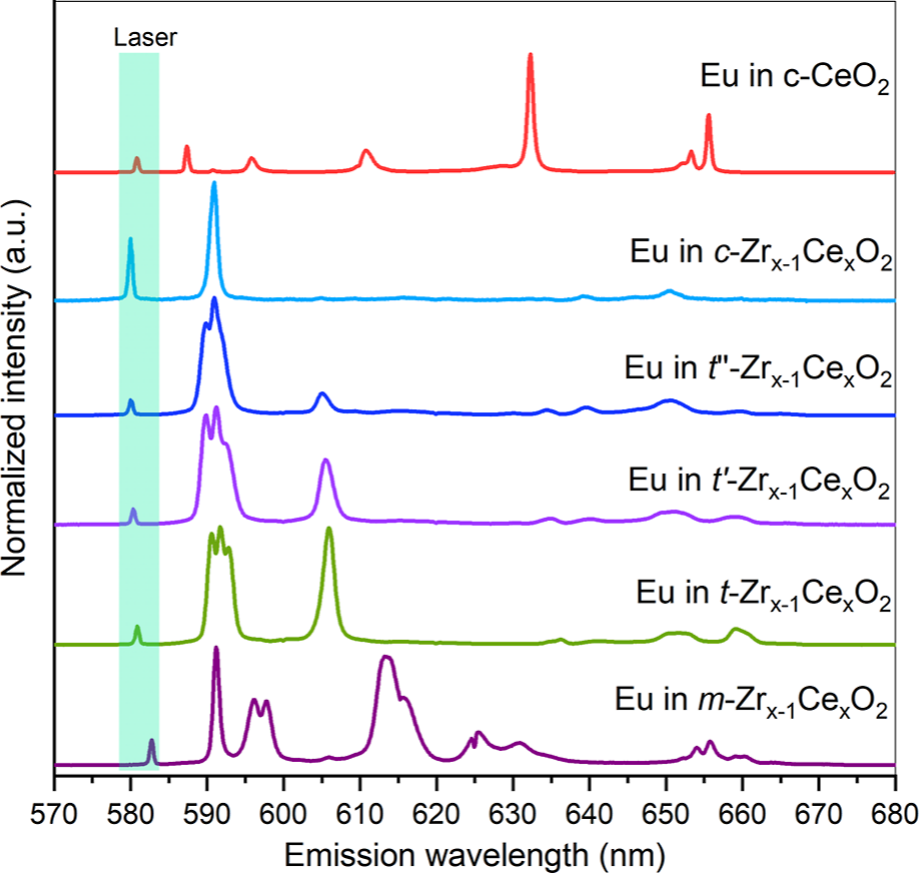
Eu^3+^ emission spectra. The spectra have been collected using different excitation wavelengths (λ_ex_) from the following samples (from bottom to top): 15 mol % Ce with λ_ex_ = 582.8 nm (m-Zr_1–*x*_Ce_*x*_O_2_), 22 mol % Ce with λ_ex_ = 580.81 nm (t-Zr_1–*x*_Ce_*x*_O_2_), 50 mol % Ce with λ_ex_ = 580.3 nm (t′-Zr_1–*x*_Ce_*x*_O2), 80 mol % Ce with λ_ex_ = 580.11 nm (t″-Zr_1–*x*_Ce_*x*_O_2_), 90 mol % Ce with λ_ex_ = 580.13 nm (c-Zr_1–*x*_Ce_*x*_O_2_) and CeO_2_ with λ_ex_ = 580.87 nm (c-CeO_2_).

The samples with the lowest Ce doping, 15 and 16 mol % Ce, exhibit a red-shifted, and low-intensity signal at approximately 582.8 nm, which is absent in all other compositions. To selectively excite this Eu^3+^ environment, the laser excitation wavelength (λ_ex_) was tuned to 582.8 nm (corresponding to the excitation peak maximum). The resulting emission spectra (Figures 8 and S14a,b) displays a 3-fold splitting of the ^5^D_0_ → ^7^F_1_ band and a 5-fold splitting of the ^7^F_2_ band, characteristic of a low-symmetry environment for the Eu^3+^ ion. The emission spectrum is consistent with published data for Eu^3+^ incorporated in a monoclinic ZrO_2_ crystal structure, and it is thus labeled as Eu in m-Zr_1–*x*_Ce_*x*_O_2_ in Figure 8.^55,57^

The SPXRD results indicate that the 15 mol % Ce sample contains a 48% monoclinic phase, which corroborates the more pronounced excitation band compared to the 16 mol % Ce sample (see Table 1 and Figure S14a,b), which only contains 17% of this phase. The following excitation band appears at around 581 nm and remains present in all samples containing 22 to 50 mol % Ce. It is highly pronounced for samples with doping concentrations below 30 mol % Ce in Figure 7. At 30 mol % Ce, this signal splits into two bands with a small shift of the peak position of only 0.5 nm. Above this concentration, the initial band gradually diminishes while the new signal becomes dominant. From the SPXRD and Raman investigations, we know that the tetragonal phase prevails between 16 and 30 mol % Ce, after which the t′ phase, i.e., the Zr_0.5_Ce_0.5_O_2_ phase, becomes predominant at 42 mol % Ce. The formation of these phases coincides with the two excitation peak signals in the luminescence measurements.

Detailed examination of the excitation spectra shows that they can be decomposed into two or three excitation peaks, depending on whether only the t-phase or both t and t′ phases are present. These spectra are shown in Figures S13 and S14 together with a short description of the broad, blue-shifted signal present in all compositions, which will not be discussed further here, as it stems from the surface, rather than the bulk structure.^27,54^ The emission spectra collected after selective excitation around 581 nm, correspond to europium in a tetragonal structure, Figure 8 (green traces). Additionally, the spectra collected after excitation at approximately 580 nm stem from the tetragonal prime structure, Figure 8 (light purple traces).

When comparing the two emission spectra, slight differences can be observed. In both cases, the ^7^F_1_ band splits into three, and a singlet ^7^F_2_ band is present. However, the ^7^F_1_ band is relatively broader, and the primary distinction lies in the ^7^F_2_ band intensity (Figure S15a). The ^7^F_2_/^7^F_1_ ratio, often referred to as the asymmetry ratio, can be calculated by integrating the emission bands corresponding to the ^7^F_2_ and ^7^F_1_ transitions in the emission spectra. A lower value of this ratio indicates a higher degree of relative symmetry in the samples.^52^ Eu^3+^ in the tetragonal phase is characterized by a ^7^F_2_/^7^F_1_ ratio of 0.72 ± 0.09. This ratio decreases significantly to 0.32 ± 0.09 for the t′ phase. This is consistent with the observed decrease of the *c*/*a*_n_ ratio from 1.018 (t) to 1.008 (t′) obtained from our SPXRD analysis (Table S2), which shows that the lattice parameters approach a cubic configuration with a higher symmetry than that of the tetragonal phase.

At 60 mol % Ce, the excitation wavelength shifts to lower values again, indicating the return of the solid solution system. However, between 70 and 80 mol % Ce the excitation spectra remain almost constant once more, differing only with respect to the half width (Figure S15b,c). To understand the phases occurring in the compositions with 60 to 80 mol % Ce, it is important to consider all findings for these compositions. The SPXRD shows a decrease in the *c*/*a*_n_ ratio to 1.003 at 60 mol % Ce, and a ratio of 1.0 between 70 and 80 mol %. The Raman analysis excluded the presence of a cubic phase in this concentration range, corroborating the presence of a tetragonal double prime phase between 70 and 80 mol % Ce. The absence of the D-band at 80 mol % Ce suggests a pure t″ phase in this composition. The emission spectra analysis provides further insights. To understand the observations in more detail, it is important to point out that the Stark splitting for Eu^3+^ in a cubic environment (Schönflies point group *O*_*h*_) should not lift the degeneracy of the ^7^F_1_ band (at approximately 590 nm) and only one signal should be visible. The ^7^F_2_ band, however, should split into a doublet. For a regular tetragonal phase (space group *P*4_2_/*nmc*, Schönflies point group *D*_4*h*_) a 2-fold and 4-fold splitting for the ^7^F_1_ and ^7^F_2_ bands, respectively are expected. For the metastable tetragonal phases, a transition from 2-fold to 1-fold splitting of the ^7^F_1_ band is a reasonable assumption. Due to the very low intensity of the ^7^F_2_ band in the 60 to 90 mol % compositions, it is not a useful transition for the identification of metastable phases. However, the ^7^F_2_/^7^F_1_ ratio is still a valid measure of phase transformations in the samples. For 60 and 70 mol % Ce, the ^7^F_1_ band shows the presence of two signals, indicative of a tetragonal phase (Figure S14g,h). For 75 and 80 mol % Ce (Figure S14i,j), the ^7^F_1_ band still shows a 2-fold splitting, but the ^7^F_2_/^7^F_1_ ratio decreases from 0.24 in the 60 and 70 mol % compositions to 0.14. In other words, a phase transformation from a phase with slightly lower symmetry (t′) to a higher symmetry phase (t″) occurs between 70 and 75 mol %, consistent with SXRD and Raman data. At 80 mol % Ce, the t″ phase is the sole phase present, and it is related to europium in a tetragonal double prime structure, Figure 8 (dark blue traces).

At 90 mol % Ce, the ^7^F_2_ band has completely vanished, coinciding with the formation of the cubic fluorite crystal structure without defects or additional oxygen displacements (Figure S14k). This is represented as europium in a cubic structure, Figure 8 (light blue traces). Interestingly, when reaching the pure CeO_2_ composition, the excitation spectrum shows a strong red-shift from 580.08 nm for 90 mol % Ce to 580.88 nm for CeO_2_. In addition, a small, second signal at 580.60 nm is visible (Figure S14l). It is known that CeO_2_ has a fluorite structure with Ce^4+^ ions surrounded by eight oxygen ions in *O*_*h*_ symmetry. However, the observed emission spectrum (Figure 8, red traces) is different from the expected fluorite structure.^58^ In the emission spectra of CeO_2_ at λ_ex_ = 580.88 nm, the intensity of the ^7^F_1_ band is much lower compared to the ^7^F_2_ band (^7^F_2_/^7^F_1_ = 7.37). This indicates an abrupt decrease in the relative symmetry around the incorporated Eu^3+^ ion. Moreover, a red-shift is indicative of a stronger crystal field effect in the pure CeO_2_ structure than in the Zr-doped cubic phase. Once Eu^3+^ replaces Ce^4+^, charge compensation is expected to maintain charge equilibrium. Previous studies have suggested two possibilities to explain the presence of defects: O^2–^ vacancies (two Eu^3+^ replacing two Ce^4+^ with the concurrent formation of one O_vac_) or interstitial Eu^3+^ (four Eu^3+^ replacing three Ce^4+^). The former mechanism, however, is the more widely accepted one.^27,59,60^ A direct coordination of an oxygen vacancy to the incorporated Eu^3+^ cation would induce a distortion of the local environment.^60^ However, the same defects are expected in the Zr_1–*x*_Ce_*x*_O_2_ compositions as well. Due to the complete absence of a ^7^F_2_ band in the Zr_0.9_Ce_0.1_O_2_ composition and the single ^7^F_1_ peak, the Eu^3+^ environment is consistent with a cubic *O*_*h*_ symmetry. This is only possible, if the vacancies are not directly coordinating with the Eu^3+^ cation, but are located elsewhere in the lattice, either coordinating with Zr^4+^ or Ce^4+^. As the spectra change drastically in the absence of Zr^4+^, in the pure CeO_2_ sample, vacancy coordination is likely to occur to Zr^4+^. In the absence of Zr^4+^, defect coordination occurs directly to Eu^3+^, with the subsequent lowering of the local symmetry. This assignment is supported by previous studies, showing coordination of oxygen vacancies in the nearest neighbor and next nearest neighbor positions to Eu^3+^ in the CeO_2_ lattice.^59,61^

In summary, the luminescence spectroscopy reveals pronounced changes in the excitation and emission spectra, as well as the ^7^F_2_/^7^F_1_ band ratio for samples with varying cerium concentration. Initially, monoclinic ZrO_2_, found in the samples with 15 and 16 mol % Ce, is characterized by an excitation peak at 582.80 nm. The emission spectra show full lifting of the degeneracy in a low symmetry environment, resulting in 3-fold and 5-fold splitting of the ^7^F_1_ and ^7^F_2_ bands, respectively. Between 20 and 50 mol % the stoichiometric phases Zr_0.8_Ce_0.2_O_2_ with t structure and Zr_0.5_C_e0.5_O_2_ with t́ structure are present. These phases are characterized by excitation spectra at 580.86 and 580.43 nm with different ^7^F_2_/^7^F_1_ ratios of 0.72 ± 0.09 and 0.32 ± 0.09. Between 60 and 80 mol % Ce, a transition from the t′ to t″ phase occurs. The t″ phase is characterized by a clearly narrower excitation spectrum at 581.15 nm and a ^7^F_2_/^7^F_1_ ratio of 0.14. In other words, a systematic decrease of the ^7^F_2_/^7^F_1_ ratio from t to t′ to t″ is obtained, speaking for a systematic increase in the relative symmetry of these phases. The sample with 90% Ce is purely cubic, with an excitation spectrum at 580.08 nm. The emission spectrum is characterized by the full absence of a ^7^F_2_ band. Finally, the CeO_2_ sample shows a more complex Eu^3+^ luminescence behavior, which includes a strong red-shift of the excitation spectrum to 580.88 nm, and a large ^7^F_2_/^7^F_1_ ratio of 7.37. The full lifting of the degeneracy of the ^7^F_2_ and ^7^F_1_ bands is evident, which can be attributed to the coordination of oxygen vacancies to the Eu^3+^ ions, with the subsequent lowering of the site-symmetry.

## Conclusions

In this study, we successfully synthesized 25 (Zr_1–*x*_Ce_*x*_O_2_) compositions by the coprecipitation route, without any detectable phase separation. The findings reveal the complex phase behavior of the ZrO_2_–CeO_2_ system, with a wide range of structures stabilized by varying the cerium content. In-depth characterization identified the presence of monoclinic, tetragonal, tetragonal prime, and double prime phases, as well as the cubic crystal structure. Furthermore, a miscibility gap was identified between 20 and 50 mol % Ce, where two stoichiometric phases, t-Zr_0.80_Ce_0.20_O_2_ and t′-Zr_0.5_Ce_0.5_O_2_, coexist. The confirmation of the metastable phase was achieved through a combination of techniques, including the SPXRD analysis, which indicated the presence of metastable phases with a *c*/*a*_n_ ratio below 1.010. Our Raman studies revealed the presence of a D-band in the compositions with a t′ structure. The HERFD-XANES analysis was essential to confirm the presence of only Ce^4+^ in the sample, indicating that the D-band can be attributed to the structural distortion induced by the oxygen displacement in the tetragonal metastable phases. Europium luminescence analysis not only identified the europium species for all crystal phases but also, together with the Raman analysis, clearly distinguished the metastable t′ and t″ phases from the cubic structure. The different excitation wavelengths evidenced the presence of different Eu^3+^ environments, with a broader ^7^F_1_ emission band potentially associated with higher defect densities, and the decreasing ^7^F_2_ intensity related to increased relative symmetry, where the absence of the ^7^F_2_ band signified the highest relative symmetry, in this case, the cubic fluorite structure. The insights gained from these results conclusively demonstrated the existence of various metastable phases with cerium incorporation, a key finding that may allow the design of high-performance materials with different applications such as in photonics or light conversion devices.

## Experimental Section

### Synthesis

The reagents were used without prior purification. The cerium-doped zirconia series was prepared according to the following procedure. First, two stock solutions were prepared by dilution of CeCl_3_·7H_2_O (Alfa Aesar) and EuCl_3_·6H_2_O (Sigma-Aldrich) in 1 mmol of HCl (ACROS, 37%) to reach the concentration of 2.763 and 0.01 M, respectively. The synthesis started with the dissolution of ZrOCl_2_·8H_2_O (0.4 g, 1.23 mmol) (Sigma-Aldrich) in 0.5 mL of 0.01 M HCl. Subsequently, appropriate amounts of Ce^3+^ and Eu^3+^ were mixed to reach the desired doping concentration of Cerium, varying from 10 mol % (0.0495 mL, 0.136 mmol) to 90 mol % (3.480 mL, 1.14 mmol), and 0.01 mol % Eu in the final ZrO_2_. The resulting solution was thereafter slowly added to 12.5% NH_4_OH (25% Sigma-Aldrich) under constant stirring, leading to instantaneous precipitation. A CeO_2_ precursor was synthesized using similar synthesis conditions, where the Ce-stock solution (0.445 mL, 1.23 mmol) was added in 0.5 mL HCl together with 0.001 mmol of EuCl_3_·6H_2_O, followed by NH_4_OH addition to induce precipitation. The resulting suspensions were kept at room temperature (25 °C) for approximately 20 h to ensure complete precipitation. Subsequently, the solid phases were separated via centrifugation, and the precipitates were washed six times with doubly deionized water (Milli-Q-grade, with a resistivity of 18.2 MΩ cm). The supernatant was checked using QUANTOFIX ammonia selective strips to confirm the removal of all ammonia residue. The samples were dried, and the powder was mortared and pressed into two pellets, each 13 mm in diameter, using a hydraulic press (PerkinElmer GmbH) at 10 Torr (1333 N/m^2^). The pellets were sintered at 1500 °C for 48 h to obtain a crystalline solid, which was manually ground to a fine powder in an agate mortar. The cooling/heating rate was 3 °C/min. According to Chevalier et al., for a grain size below 500 nm, the estimated time for diffusion to homogenize a zirconia solid phase consisting of Zr, Y, and O, is 24 h at 1500 °C. Since yttrium has a bigger ionic radius than cerium and the scattering domain sizes in all our samples are smaller (<162 nm, see Table S2), we can assume that the diffusion will be faster and homogeneous solids are obtained during calcination.^62^

### Synchrotron Powder X-ray Diffraction

The SPXRD measurements were performed at the XRD-1 station of the ROBL beamline at the European Synchrotron Radiation Facility, ESRF, Grenoble, France.^63^ The X-ray beam was focused to a size of 200 × 200 μm^2^. The data were collected in transmission mode with an Eiger CdTe 500k detector (Dectris) and integrated using a specific Python code based on PyFAI routines adapted to treat a series of images collected as a function of 2θ.^64^ The wavelength of the synchrotron radiation source was 0.774901 Å for samples ranging from 10 to 70 mol % Ce, and 0.724901 Å for the remaining ones. The horizontal polarization was set to 0.950 in both cases. The samples were analyzed within a glass capillary with a 0.3 mm diameter. For data comparison, the Bragg equation was applied to all the results to convert 2θ to the interplanar distance, which was subsequently converted back to 2θ with the same X-ray wavelength of 0.774901 Å for all samples. The energy was calibrated against a Y metal foil at the Y K-edge of 17038.0 eV/λ = 0.727692 Å or 16000.0 eV/λ = 0.774901 Å. This wavelength was used for the measurements. The Rietveld refinement was performed with PDXL2 software version 2.6.1.2. The crystallite size was determined by the Debye–Scherrer equation: *D* = *k*·λ/β·cosθ where *D* is the scattering domain size which is assumed to give the average crystallite size, k is the shape factor, which was determined as 0.9 in previous studies, λ is the X-ray wavelength, in this case 0.0774901 nm, β is the angular line width of the full width at half-maximum (fwhm) of the intensity, and θ is the Bragg angle.^57^

### Raman Spectroscopy

The 2D Raman measurements were conducted using a HORIBA Jobin Yvon LabRAM Aramis Vis Raman microscope. A HeNe laser was employed without any filters, using a hole and slit size of 200 μm, and a grating with 1200 lines/mm. All spectra were recorded using an exposure time of 15 s (s) and 5 accumulations. No signs of laser-induced damage or spectral changes were observed when varying the excitation wavelength (Figure S11, left), exposure time, or grating settings (Figure S11, right).

### High Energy Resolution Fluorescence Detected X-ray Absorption Near Edge Structure

The HERFD-XANES measurements were conducted at room temperature at the ROBL beamline at the European Synchrotron Radiation Facility (ESRF). The samples were mounted between two layers of Kapton foil. The HERFD-XANES data at the Ce L_3_-edge were recorded in the energy range of 5710–5779 eV with a typical acquisition time of 4 min per spectrum using an X-ray emission spectrometer equipped with five Ge(331) analyzer crystal.

### Luminescence Spectroscopy

Eu^3+^ luminescence spectroscopic investigations were conducted at 9 K using a helium-refrigerated cryostat (Cryophysics CCS 100, Cryophysics). The laser beam was produced by a Surelite SLI-20 532 nm pump laser (Continuum) fixed with a tunable dye laser (NarrowScanK) equipped with a Rhodamine 6G/Rhodamine B dye mixture. The luminescence emission was detected using an ICCD camera, IStar340, with a rectangular CCD sensor of 2.048 × 512 × 13.5 μm pixels (Quantum Design). For all spectra acquisitions, the parameters used for gate delay and gate width were 1 μs and 10 ms, respectively. The Eu^3+^ excitation spectra were recorded with a 600 lines/mm grating between 572 and 584 nm using 100 accumulations per spectrum. For emission spectra at selected excitation wavelengths, 2500 accumulations were collected. In the collected emission spectra transitions from the ^5^D_0_ excited state to the *J* = 0–2 levels of the ^7^F ground term were recorded. The ^5^D_0_ → ^7^F_0_ transition is forbidden, but can occasionally be observed between 575 and 585 nm. The ^5^D_0_ → ^7^F_1_ magnetic dipole transitions occur in the 585–600 nm range, while the ^5^D_0_ → ^7^F_2_ electric dipole transitions, which are sensitive to the symmetry of the environment, occur around 610–630 nm. The multiplicity of peaks in the spectra is associated with the splitting of the Eu^3+^ 4f shell.^52^

## Supporting Information Available

Experimental results from Synchrotron X-ray Diffraction including diffractograms, details on the Rietveld refinement, tetragonality and average scattering domain size calculations, z coordinates in all sample compositions, trends in fwhm and diffraction reflection positions across samples, experimental results from Raman investigations, including Raman spectra and the identification and assignment of Raman vibrational modes, Lorentzian fitting results of selected samples compositions, trends in fwhm and Raman band positions, Raman spectra at different laser wavelengths and grating settings, and experimental results from luminescence spectroscopic investigations, including normalized excitation spectra, description of Lorentzian fitting of excitation spectra and corresponding fits, recorded excitation and emission spectra, and trends in excitation peak positions, fwhm and ^7^F_2_/^7^F_1_ band ratios (PDF)

## References

[ref1] Gionco C.; Paganini M. C.; Chiesa M.; Maurelli S.; Livraghi S.; Giamello E. Cerium doped zirconium dioxide as a potential new photocatalytic material. The role of the preparation method on the properties of the material. Appl. Catal., A 2015, 504, 338–343. 10.1016/j.apcata.2015.02.021.

[ref2] Peng Y.; Si X.-L.; Shang C.; Liu Z.-P. Abundance of Low-Energy Oxygen Vacancy Pairs Dictates the Catalytic Performance of Cerium-Stabilized Zirconia. J. Am. Chem. Soc. 2024, 146 (15), 10822–10832. 10.1021/jacs.4c01285. 38591182

[ref3] Sayle D. C.; Maicaneanu S. A.; Watson G. W. Atomistic Models for CeO_2_(111), (110), and (100) Nanoparticles Supported on Yttrium-Stabilized Zirconia. J. Am. Chem. Soc. 2002, 124 (38), 11429–11439. 10.1021/ja020657f. 12236757

[ref4] Livraghi S.; Paganini M. C.; Giamello E.; Di Liberto G.; Tosoni S.; Pacchioni G. Formation of Reversible Adducts by Adsorption of Oxygen on Ce–ZrO_2_: An Unusual η2 Ionic Superoxide. J. Phys. Chem. C 2019, 123 (44), 27088–27096. 10.1021/acs.jpcc.9b08615.

[ref5] Tu H.; Liu X.; Yu Q. Synthesis and characterization of scandia ceria stabilized zirconia powders prepared by polymeric precursor method for integration into anode-supported solid oxide fuel cells. J. Power Sources 2011, 196 (6), 3109–3113. 10.1016/j.jpowsour.2010.11.108.

[ref6] Tao S.; Irvine J. T. S. A redox-stable efficient anode for solid-oxide fuel cells. Nat. Mater. 2003, 2 (5), 320–323. 10.1038/nmat871. 12692533

[ref7] Tsuchiya M.; Lai B.-K.; Ramanathan S. Scalable nanostructured membranes for solid-oxide fuel cells. Nat. Nanotechnol. 2011, 6 (5), 282–286. 10.1038/nnano.2011.43. 21460827

[ref8] Lamas D. G.; Lascalea G. E.; Juárez R. E.; Djurado E.; Pérez L.; Walsöe de Reca N. E. Metastable forms of the tetragonal phase in compositionally homogeneous, nanocrystalline zirconia–ceria powders synthesised by gel-combustion. J. Mater. Chem. 2003, 13 (4), 904–910. 10.1039/b210500b.

[ref9] Monte R. D.; Kašpar J. Nanostructured CeO_2_–ZrO_2_ mixed oxides. J. Mater. Chem. 2005, 15 (6), 633–648. 10.1039/B414244F.

[ref10] Varez A.; Garcia-Gonzalez E.; Jolly J.; Sanz J. Structural characterization of Ce_1–x_Zr_x_O_2_ (0 ≤ *x* ≤ 1) samples prepared at 1650°C by solid state reaction: A combined TEM and XRD study. J. Eur. Ceram. Soc. 2007, 27 (13), 3677–3682. 10.1016/j.jeurceramsoc.2007.02.014.

[ref11] Verdi C.; Karsai F.; Liu P.; Jinnouchi R.; Kresse G. Thermal transport and phase transitions of zirconia by on-the-fly machine-learned interatomic potentials. npj Comput. Mater. 2021, 7 (1), 156 10.1038/s41524-021-00630-5.

[ref12] Zhang F.; Chen C.-H.; Hanson J. C.; Robinson R. D.; Herman I. P.; Chan S.-W. Phases in Ceria–Zirconia Binary Oxide (1–x)CeO_2_–xZrO_2_ Nanoparticles: The Effect of Particle Size. J. Am. Ceram. Soc. 2006, 89 (3), 1028–1036. 10.1111/j.1551-2916.2005.00788.x.

[ref13] Sanjuán M. L.; Oliete P. B.; Várez A.; Sanz J. The role of Ce reduction in the segregation of metastable phases in the ZrO_2_–CeO_2_ system. J. Eur. Ceram. Soc. 2012, 32 (3), 689–696. 10.1016/j.jeurceramsoc.2011.10.015.

[ref14] Yashima M. Crystal Structures of the Tetragonal Ceria–Zirconia Solid Solutions Ce_x_Zr_1–x_O_2_ through First Principles Calculations (0 ≤ *x* ≤ 1). J. Phys. Chem. C 2009, 113 (29), 12658–12662. 10.1021/jp9024156.

[ref15] Svitlyk V.; Weiss S.; Hennig C. Immobilization of radiotoxic elements with Y-stabilized zirconia: The thorium case. J. Am. Ceram. Soc. 2022, 105 (9), 5975–5983. 10.1111/jace.18543.

[ref16] Matsui K.; Yoshida H.; Ikuhara Y. Nanocrystalline, Ultra-Degradation-Resistant Zirconia: Its Grain Boundary Nanostructure and Nanochemistry. Sci. Rep. 2014, 4 (1), 4758 10.1038/srep04758. 24755733 PMC3996460

[ref17] Knipe K.; Manero A.; Siddiqui S. F.; Meid C.; Wischek J.; Okasinski J.; Almer J.; Karlsson A. M.; Bartsch M.; Raghavan S. Strain response of thermal barrier coatings captured under extreme engine environments through synchrotron X-ray diffraction. Nat. Commun. 2014, 5 (1), 4559 10.1038/ncomms5559. 25078347

[ref18] Wang K.; Zhao M.; Ren X.; Pan W. High temperature mechanical properties of zirconia metastable t’-Phase degraded yttria stabilized zirconia. Ceram. Int. 2019, 45 (14), 17376–17381. 10.1016/j.ceramint.2019.05.297.

[ref19] Devaiah D.; Reddy L. H.; Park S.-E.; Reddy B. M. Ceria–zirconia mixed oxides: Synthetic methods and applications. Catal. Rev. 2018, 60 (2), 177–277. 10.1080/01614940.2017.1415058.

[ref20] Berguzinov A.; Kozlovskiy A.; Khametova A. A.; Shlimas D. I. Synthesis, Phase Transformations and Strength Properties of Nanostructured (1 – x)ZrO2 – xCeO2 Composite Ceramics. Nanomaterials 2022, 12 (12), 1979 10.3390/nano12121979. 35745319 PMC9229295

[ref21] Duwez P. O.; Odell F. Phase Relationships in the System Zirconia—Ceria. J. Am. Ceram. Soc. 1950, 33 (9), 274–283. 10.1111/j.1151-2916.1950.tb12798.x.

[ref22] Garvie R. C.; Hannink R. H.; Pascoe R. T. Ceramic steel?. Nature 1975, 258 (5537), 703–704. 10.1038/258703a0.

[ref23] Rejab N. A.; Azhar A. Z. A.; Ratnam M. M.; Ahmad Z. A. The effects of CeO_2_ addition on the physical, microstructural and mechanical properties of yttria stabilized zirconia toughened alumina (ZTA). Int. J. Refract. Met. Hard Mater. 2013, 36, 162–166. 10.1016/j.ijrmhm.2012.08.010.

[ref24] Yashima M.; Arashi H.; Kakihana M.; Yoshimura M. Raman Scattering Study of Cubic–Tetragonal Phase Transition in Zr_1–x_Ce_x_O_2_ Solid Solution. J. Am. Ceram. Soc. 1994, 77 (4), 1067–1071. 10.1111/j.1151-2916.1994.tb07270.x.

[ref25] Shukla S.; Seal S. Mechanisms of room temperature metastable tetragonal phase stabilisation in zirconia. Int. Mater. Rev. 2005, 50 (1), 45–64. 10.1179/174328005X14267.

[ref26] Kaduk J. A.; Billinge S. J. L.; Dinnebier R. E.; Henderson N.; Madsen I.; Černý R.; Leoni M.; Lutterotti L.; Thakral S.; Chateigner D. Powder diffraction. Nat. Rev. Methods Primers 2021, 1 (1), 77 10.1038/s43586-021-00074-7.

[ref27] Eibl M.; Shaw S.; Prieur D.; Rossberg A.; Wilding M. C.; Hennig C.; Morris K.; Rothe J.; Stumpf T.; Huittinen N. Understanding the local structure of Eu^3+^- and Y^3+^-stabilized zirconia: insights from luminescence and X-ray absorption spectroscopic investigations. J. Mater. Sci. 2020, 55 (23), 10095–10120. 10.1007/s10853-020-04768-3.

[ref28] Uvarov V. The influence of X-ray diffraction pattern angular range on Rietveld refinement results used for quantitative analysis, crystallite size calculation and unit-cell parameter refinement. J. Appl. Crystallogr. 2019, 52 (2), 252–261. 10.1107/S1600576719000621.

[ref29] Zhao P.; Lu L.; Liu X.; De la Torre A.; Cheng X. Error Analysis and Correction for Quantitative Phase Analysis Based on Rietveld-Internal Standard Method: Whether the Minor Phases Can Be Ignored?. Crystals 2018, 8 (3), 110 10.3390/cryst8030110.

[ref30] Vailionis A. 7-The effects of strain on crystal structure and properties during epitaxial growth of oxides. In Epitaxial Growth of Complex Metal Oxides; Woodhead Publishing, 2015; pp 175–207.

[ref31] Tani E.; Yoshimura M.; Somiya S. Revised Phase Diagram of the System ZrO_2_-CeO_2_ Below 1400°C. J. Am. Ceram. Soc. 1983, 66 (7), 506–510. 10.1111/j.1151-2916.1983.tb10591.x.

[ref32] Yashima M.; Takashina H.; Kakihana M.; Yoshimura M. Low-Temperature Phase Equilibria by the Flux Method and the Metastable–Stable Phase Diagram in the ZrO_2_–CeO_2_ System. J. Am. Chem. Soc. 1994, 77 (7), 1869–1874. 10.1111/j.1151-2916.1994.tb07064.x.

[ref33] Duran P.; Gonzalez M.; Moure C.; Jurado J. R.; Pascual C. A new tentative phase equilibrium diagram for the ZrO_2_-CeO_2_ system in air. J. Mater. Sci. 1990, 25 (12), 5001–5006. 10.1007/BF00580121.

[ref34] Abbas Z.; Surendran M.; Anjana P. A.; Jidev P. K.; Dasari H.; Sudhakar Naidu N.; Anandhan S.; Bhat K.; Bhaskar Babu G.; Prasad Dasari H. Solubility Limits of Ceria-Zirconia-Lanthana Solid-Solutions. Mater. Today: Proc. 2017, 4 (9), 9360–9364. 10.1016/j.matpr.2017.06.185.

[ref35] He J.; Yao P.; Qiu J.; Zhang H.; Jiao Y.; Wang J.; Chen Y. Enhancement effect of oxygen mobility over Ce_0.5_Zr_0.5_O_2_ catalysts doped by multivalent metal oxides for soot combustion. Fuel 2021, 286, 119359 10.1016/j.fuel.2020.119359.

[ref36] Nagai Y.; Yamamoto T.; Tanaka T.; Yoshida S.; Nonaka T.; Okamoto T.; Suda A.; Sugiura M. Local structure analyses of Ce_0.5_Zr_0.5_O_2_ mixed oxides by XAFS. J. Synchrotron Radiat. 2001, 8 (2), 616–618. 10.1107/s0909049500017520. 11512871

[ref37] Lemaux S.; Bensaddik A.; van der Eerden A. M. J.; Bitter J. H.; Koningsberger D. C. Understanding of Enhanced Oxygen Storage Capacity in Ce_0.5_Zr_0.5_O_2_: The Presence of an Anharmonic Pair Distribution Function in the Zr–O_2_ Subshell as Analyzed by XAFS Spectroscopy. J. Phys. Chem. B 2001, 105 (21), 4810–4815. 10.1021/jp003111t.

[ref38] National Institute of Standards & Technology Standard Reference Material 674b, X-Ray Powder Diffraction Intensity Set (Quantitative Powder Diffraction Standard); National Institute of Standards & Technology, 2017.

[ref39] Han X. X.; Rodriguez R. S.; Haynes C. L.; Ozaki Y.; Zhao B. Surface-enhanced Raman spectroscopy. Nat. Rev. Methods Primers 2022, 1 (1), 87 10.1038/s43586-021-00083-6.

[ref40] Xu Y.; Wang F.; Liu X.; Liu Y.; Luo M.; Teng B.; Fan M.; Liu X. Resolving a Decade-Long Question of Oxygen Defects in Raman Spectra of Ceria-Based Catalysts at Atomic Level. J. Phys. Chem. C 2019, 123 (31), 18889–18894. 10.1021/acs.jpcc.9b00633.

[ref41] Efaw C. M.; Vandegrift J. L.; Reynolds M.; McMurdie S.; Jaques B. J.; Hu H.; Xiong H.; Hurley M. F. Characterization of zirconium oxides part I: Raman mapping and spectral feature analysis. Nucl. Mater. Energy 2019, 21, 100707 10.1016/j.nme.2019.100707.

[ref42] Quintard P. E.; Barbéris P.; Mirgorodsky A. P.; Merle-Méjean T. Comparative Lattice-Dynamical Study of the Raman Spectra of Monoclinic and Tetragonal Phases of Zirconia and Hafnia. J. Am. Ceram. Soc. 2002, 85 (7), 1745–1749. 10.1111/j.1151-2916.2002.tb00346.x.

[ref43] Kim B.-K.; Hamaguchi H.-o. Mode Assignments of the Raman Spectrum of Monoclinic Zirconia by Isotopic Exchange Technique. Phys. Status Solidi B 1997, 203 (2), 557–563. 10.1002/1521-3951(199710)203:2<557::AID-PSSB557>3.0.CO;2-C.

[ref44] Vlaic G.; Fornasiero P.; Geremia S.; Kašpar J.; Graziani M. Relationship between the Zirconia-Promoted Reduction in the Rh-Loaded Ce_0.5_Zr_0.5_O_2_ Mixed Oxide and the Zr–O Local Structure. J. Catal. 1997, 168 (2), 386–392. 10.1006/jcat.1997.1644.

[ref45] Naumenko A. P.; Berezovska N.; Biliy M. M.; Shevchenko O. V. Vibrational Analysis and Raman Spectra of Tetragonal Zirconia. Phys. Chem. Org. Solid State 2008, 9 (1), 121–125.

[ref46] Fornasiero P.; Speghini A.; Di Monte R.; Bettinelli M.; Kašpar J.; Bigotto A.; Sergo V.; Graziani M. Laser-Excited Luminescence of Trivalent Lanthanide Impurities and Local Structure in CeO_2_–ZrO_2_ Mixed Oxides. Chem. Mater. 2004, 16 (10), 1938–1944. 10.1021/cm035370b.

[ref47] Toloshniak T.; Guhel Y.; Bernard J.; Besq A.; Marinel S.; Boudart B. Impact of microwave annealing on CeO_2_ thin films sputtered on (111) Si. Mater. Res. Bull. 2015, 70, 712–718. 10.1016/j.materresbull.2015.05.041.

[ref48] Costantini J.-M.; Miro S.; Touati N.; Binet L.; Wallez G.; Lelong G.; Guillaumet M.; Weber W. J. Defects induced in cerium dioxide single crystals by electron irradiation. J. Appl. Phys. 2018, 123 (2), 025901 10.1063/1.5007823.

[ref49] Kvashnina K. O.; Scheinost A. C. A. Johann-type X-ray emission spectrometer at the Rossendorf beamline. J. Synchrotron Radiat. 2016, 23 (3), 836–841. 10.1107/S1600577516004483. 27140166

[ref50] Kvashnina K. O.; Butorin S. M.; Glatzel P. Direct study of the f-electron configuration in lanthanide systems. J. Anal. At. Spectrom. 2011, 26 (6), 1265–1272. 10.1039/c0ja00142b.

[ref51] Kvashnina K. O. Electronic-Structure Interpretation: How Much Do We Understand Ce L_3_ XANES?. Chem.—Eur. J. 2024, 30 (46), e202400755 10.1002/chem.202400755. 38860741

[ref52] Binnemans K. Interpretation of europium(III) spectra. Coord. Chem. Rev. 2015, 295, 1–45. 10.1016/j.ccr.2015.02.015.

[ref53] Dwaraka Viswanath C. S.; Venkata Krishnaiah K.; Jayasankar C. K. Luminescence properties of europium doped oxyfluorosilicate glasses for visible light devices. Opt. Mater. 2018, 83, 348–355. 10.1016/j.optmat.2018.05.057.

[ref54] Montini T.; Speghini A.; Rogatis L. D.; Lorenzut B.; Bettinelli M.; Graziani M.; Fornasiero P. Identification of the Structural Phases of Ce_x_Zr_1–x_O_2_ by Eu(III) Luminescence Studies. J. Am. Chem. Soc. 2009, 131 (36), 13155–13160. 10.1021/ja905158p. 19673522

[ref55] Smits K.; Grigorjeva L.; Millers D.; Sarakovskis A.; Opalinska A.; Fidelus J. D.; Lojkowski W. Europium doped zirconia luminescence. Opt. Mater. 2010, 32 (8), 827–831. 10.1016/j.optmat.2010.03.002.

[ref56] Thorat A. V.; Ghoshal T.; Carolan P.; Holmes J. D.; Morris M. A. Defect Chemistry and Vacancy Concentration of Luminescent Europium Doped Ceria Nanoparticles by the Solvothermal Method. J. Phys. Chem. C 2014, 118 (20), 10700–10710. 10.1021/jp410213n.

[ref57] Opitz L.; Hübner R.; Shams Aldin Azzam S.; Gilson S. E.; Finkeldei S. C.; Huittinen N. Investigations towards incorporation of Eu^3+^ and Cm^3+^ during ZrO_2_ crystallization in aqueous solution. Sci. Rep. 2023, 13 (1), 12276 10.1038/s41598-023-39143-0. 37507431 PMC10382555

[ref58] Kumar A.; Babu S.; Karakoti A. S.; Schulte A.; Seal S. Luminescence Properties of Europium-Doped Cerium Oxide Nanoparticles: Role of Vacancy and Oxidation States. Langmuir 2009, 25 (18), 10998–11007. 10.1021/la901298q. 19735149

[ref59] Li T.; Li P.; Wang Z.; Xu S.; Bai Q.; Yang Z. Coexistence phenomenon of Ce^3+^–Ce^4+^ and Eu^2+^–Eu^3+^ in Ce/Eu co-doped LiBaB_9_O_15_ phosphor: luminescence and energy transfer. Phys. Chem. Chem. Phys. 2017, 19 (5), 4131–4138. 10.1039/C6CP07494D. 28111664

[ref60] Liu X.; Chen S.; Wang X. Synthesis and photoluminescence of CeO_2_:Eu^3+^ phosphor powders. J. Lumin. 2007, 127 (2), 650–654. 10.1016/j.jlumin.2007.03.014.

[ref61] Tiseanu C.; Parvulescu V. I.; Sanchez-Dominguez M.; Boutonnet M. Temperature induced conversion from surface to “bulk” sites in Eu^3+^-impregnated CeO_2_ nanocrystals. J. Appl. Phys. 2012, 112 (1), 013521 10.1063/1.4730609.

[ref62] Chevalier J.; Gremillard L.; Virkar A. V.; Clarke D. R. The Tetragonal-Monoclinic Transformation in Zirconia: Lessons Learned and Future Trends. J. Am. Ceram. Soc. 2009, 92 (9), 1901–1920. 10.1111/j.1551-2916.2009.03278.x.

[ref63] Scheinost A. C.; Claussner J.; Exner J.; Feig M.; Findeisen S.; Hennig C.; Kvashnina K. O.; Naudet D.; Prieur D.; Rossberg A.; Schmidt M.; Qiu C.; Colomp P.; Cohen C.; Dettona E.; Dyadkin V.; Stumpf T. ROBL-II at ESRF: a synchrotron toolbox for actinide research. J. Synchrotron Radiat. 2021, 28 (1), 333–349. 10.1107/s1600577520014265. 33399586 PMC7842221

[ref64] Kieffer J.; Valls V.; Blanc N.; Hennig C. New tools for calibrating diffraction setups. J. Synchrotron Radiat. 2020, 27 (2), 558–566. 10.1107/S1600577520000776. 32153298 PMC7842211

