# Supplementary material for: Exploring Metastable Phases in Cerium-Doped Zirconia: Insights from X-ray Diffraction, Raman, X-ray Absorption, and Luminescence Spectroscopy
Source: Inorg Chem. 2025 May 8;64(19):9670–83. doi: 10.1021/acs.inorgchem.5c00865 (PMC12093295; doi:10.1021/acs.inorgchem.5c00865)
Supplement: Supplementary file 1 — ic5c00865_si_001.pdf [file ic5c00865_si_001.pdf]

Supporting Information

Exploring metastable phases in cerium-doped zirconia: Insights from X-ray diffraction, Raman, X-ray absorption, and luminescence spectroscopy

Luiza B. F. dos Santos[1,2], Volodymyr Svitlyk[3], Selina Richter[1], Christoph Hennig[1,3], Katharina Müller[1], Elena F. Bazarkina[1,3], Kristina O. Kvashnina[1,3], Thorsten Stumpf[1], Nina Huittinen[1,2]*

[1]Institute of Resource Ecology, Helmholtz-Zentrum Dresden-Rossendorf, Bautzner Landstraße 400, 01328 Dresden, Germany

[2]Institute of Chemistry and Biochemistry, Freie Universität Berlin, Fabeckstraße 34-36, 14195 Berlin, Germany

[3]The Rossendorf Beamline (BM20), CS40220, European Synchrotron Radiation Facility, 38043 Grenoble Cedex 9, France

*corresponding author: nina.huittinen@fu-berlin.de

This Supporting Information (SI) contains 15 figures and 2 tables

Table of Contents

Synchrotron Powder X-Ray Diffraction 3

Figure S1: SPXRD diffractograms of all $Zr_{1-x}Ce_xO_2$ compositions (0.1 ≤ x ≤ 1.0). 3

Rietveld refinement 4

Table S1: Synthesis parameters of the samples A, B, and C. 4

Figure S2: SPXRD diffractograms of the original 10 mol% Ce-doped zirconia sample and the samples A, B, and C (top). Magnification of 13.75-15.8° 2θ range of the SPXRD diffractograms (bottom). 5

Table S2: Calculated tetragonality and average scattering domain size determined by the Debye-Scherrer equation. 6

Figure S3: Z coordinates for the oxygen ions in $Zr_{1-x}Ce_xO_2$ (0.1 ≤ x ≤ 1.0). 7

Figure S4: (a) FWHM and peak positions for the t', t'', and c peak in $Zr_{1-x}Ce_xO_2$ (0.18 ≤ x ≤ 1.0); (b) Normalized tetragonal metastable peak; (c) Normalized tetragonal peak of SPXRD diffractograms in $Zr_{1-x}Ce_xO_2$ (0.18 ≤ x ≤ 0.50); (d) Low 2θ and (e) High 2θ of SPXRD diffractograms in $Zr_{1-x}Ce_xO_2$ (0.18 ≤ x ≤ 1.0). 8

Raman and HERFD-XANES 9

Figure S5: Raman spectra of 10, 42, and 90 mol% cerium-doped zirconia, with band assignment from the lowest to the highest frequency. 9

Figure S6: Raman spectra of all the synthesized $Zr_{1-x}Ce_xO_2$ (0.1 ≤ x ≤ 1.0) compositions. 9

Figure S7: Raman spectra of 50 and 60 mol% Ce-doped zirconia without europium incorporation. 10

Figure S8: Raman spectra of 30, 42, and 70 mol% cerium-doped zirconia. 10

Figure S9: Lorentzian fitting of Raman peaks for the $Zr_{1-x}Ce_xO_2$ (0.65 ≤ x ≤ 1.0) compositions. 11

Figure S10: (a) FWHM and band position of the F_{2g} vibration mode in $Zr_{1-x}Ce_xO_2$ (0.65 ≤ x ≤ 1.0); (b) Raman spectra of $Zr_{1-x}Ce_xO_2$ (0.75 ≤ x ≤ 1.0). 12

Figure S11: Raman spectra of the $Zr_{0.82}Ce_{0.18}O_2$ sample acquired using two different laser wavelengths (left), showing no significant variation in spectral features. Repeated measurements at the same sample spot using the HeNe laser under varying grating settings (right) demonstrate the stability of the spectra and indicate the absence of laser-induced modifications. 12

Luminescence Spectroscopy 13

Figure S12: Excitation luminescence spectra of $Zr_{1-x}Ce_xO_2$ (x =, 0.15, 0.16, 0.22, 0.30, 0.42, 0.50, 0.60, 0.70, 0.75, 0.80, 0.90, and 1.0). 13

Decomposition of excitation spectra 13

Figure S13: Excitation and respective luminescence emission spectra of Eu^{3+} in $Zr_{0.7}Ce_{0.3}O_2$. 13

Figure S14: Excitation spectrum (left) and emission spectra collected at varying $\lambda_{excitation}$ (right) of synthesized $Zr_{1-x}Ce_xO_2$ (x = 0.15 (a), 0.16 (b), 0.22 (c), 0.30 (d), 0.42 (e), 0.50 (f), 0.60 (g), 0.70 (h), 0.75 (i), 0.80 (j), 0.90 (k), and 1.0(l)) compositions. 19

Figure S15: (a) Relative symmetry determined by the $^7F_2/^7F_1$ band ratio; (b) Peak position; and (c) FWHM of the excitation peak of $Zr_{1-x}Ce_xO_2$ (x = 0.15, 0.16, 0.22, 0.30, 0.42, 0.50, 0.60, 0.70, 0.75, 0.80, and 0.90). 20

References 21

Synchrotron Powder X-Ray Diffraction

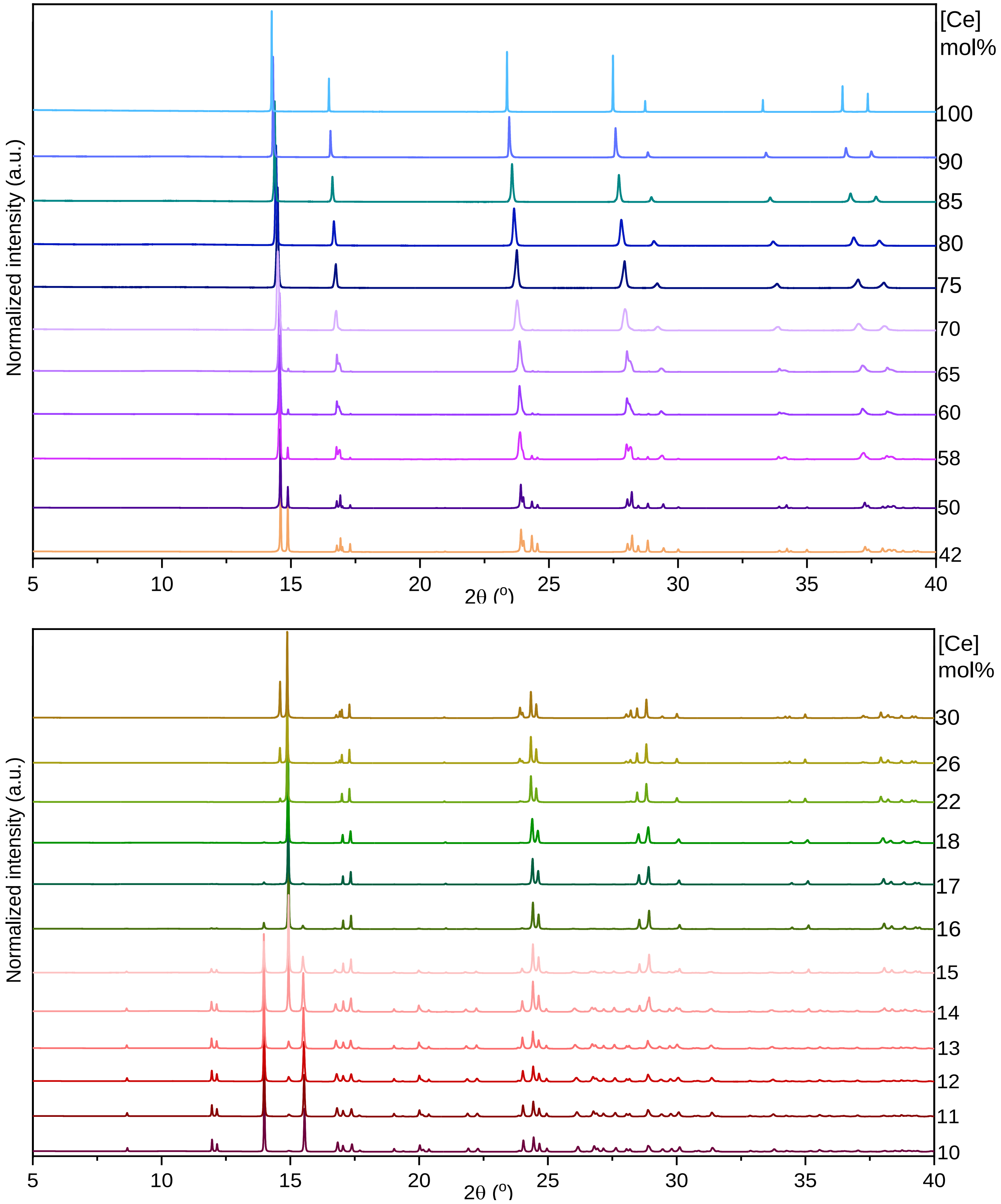

Figure S1: SPXRD diffractograms of all $Zr_{1-x}Ce_xO_2$ compositions ($0.1 \leq x \leq 1.0$).

Rietveld refinement

Rietveld refinement is a powerful tool that involves fitting a theoretical diffraction pattern or experimental data from a database to the diffraction pattern investigated, allowing for precise determination of the atomic positions, unit cell parameters, and any other structural parameters [1-3]. Mathematically, one of the parameters analyzed is the goodness of fit (s); when close to 1, the model can be considered reasonable and the result fits with the database [3]. The Rietveld refinements were performed with the Rigaku PDXL2 software version 2.6.1.2. The ICDD or COD database reference patterns for the monoclinic (m) (ICDD 40134344), tetragonal (t) (COD 1540416), tetragonal prime (t'), and double prime (t'') (ICDD 40167932), and cubic (c) (ICDD 00-028-0271) phases were used in the refinements.

To understand why the goodness of fit (s) in the samples with Ce concentrations between 10 and 14 mol% constantly yielded poor values of above 2.3, a detailed investigation was undertaken to understand the reasons for the asymmetric peaks, which were specifically observed in the monoclinic phases. Therefore, three additional syntheses were conducted, with a dopant concentration of 10 mol% Ce in all samples, while varying the synthesis parameters summarized in Table S1, including the europium addition and the pre-calcination steps. Figure S2 presents the SPXRD diffractograms, showing all peaks at the same 2θ values. However, the diffractograms of samples A and C, both synthesized without europium, exhibit symmetric peaks upon closer inspection. Conversely, sample B and the original 10 mol% Ce sample, both display asymmetric peaks, indicating their association with europium-containing samples. Even at low dopant concentrations, europium causes a distortion effect in the monoclinic phase, where due to the low cerium concentration, the cell volume is smaller in comparison to the tetragonal phase. The significant disparity in ionic radii between europium (1.066 Å) and zirconium (0.84 Å), induces a lattice disorder, leading to asymmetric peaks in the diffraction pattern of the monoclinic phase [4].

Table S1: Synthesis parameters of the samples A, B, and C.

	Eu^{3+} incorporated	Pre-calcination at 600°C for 2 hours
10 mol% Ce	Yes	No
Sample A	No	No
Sample B	Yes	Yes
Sample C	No	Yes

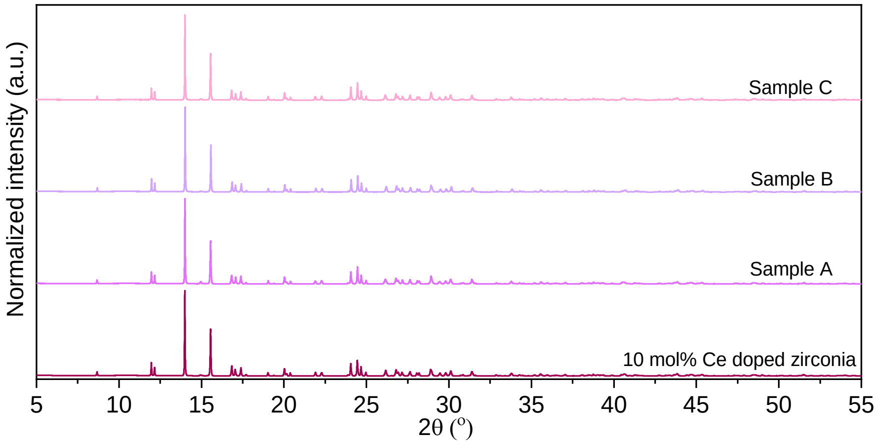

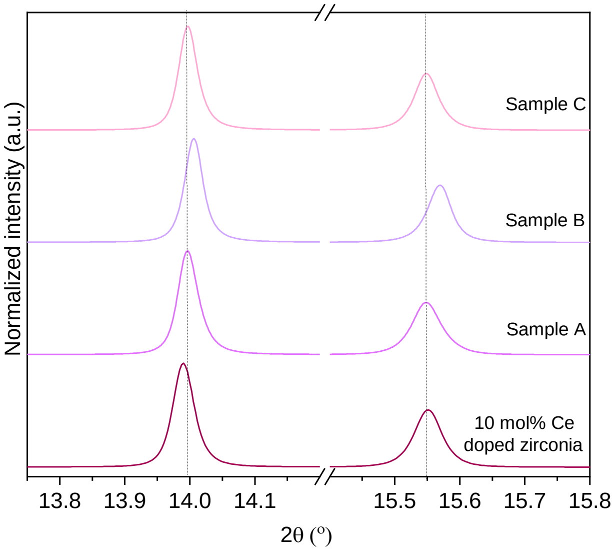

Figure S2: SPXRD diffractograms of the original 10 mol% Ce-doped zirconia sample and the samples A, B, and C (top). Magnification of 13.75-15.8° 2θ range of the SPXRD diffractograms (bottom).

Table S2: Calculated tetragonality and average scattering domain size determined by the Debye-Scherrer equation.

[Ce] mol%	tetragonality						Scattering domain size (nm)			
	t phase of $Zr_{1-x}Ce_xO_2$ solid solutions and $Zr_{0.80}Ce_{0.20}O_2$		t' phase of $Zr_{1-x}Ce_xO_2$ solid solutions and $Zr_{0.5}Ce_{0.5}O_2$		t'' phase of $Zr_{1-x}Ce_xO_2$ solid solutions		D(m)	D(t)	D(t'')	D(c)
	c/a	$c/a_{(n)}$	c/a	$c/a_{(n)}$	c/a	$c/a_{(n)}$				
10	-	-	-	-	-	-	103.4	-	-	-
11	-	-	-	-	-	-	100.4	-	-	-
12	-	-	-	-	-	-	96.9	44.0	-	-
13	-	-	-	-	-	-	104.1	53.7	-	-
14	1.439	1.018	-	-	-	-	100.3	86.8	-	-
15	1.439	1.018	-	-	-	-	88.7	96.9	-	-
16	1.439	1.018	-	-	-	-	81.3	97.4	-	-
17	1.439	1.018	-	-	-	-	62.9	95.4	-	-
18	1.439	1.018	1.424	1.007	-	-	-	78.9	-	-
22	1.438	1.017	1.425	1.007	-	-	-	104.9	81.2	-
26	1.438	1.017	1.425	1.008	-	-	-	114.3	97.2	-
30	1.438	1.017	1.426	1.008	-	-	-	113.0	97.9	-
42	1.439	1.018	1.426	1.008	-	-	-	113.2	103.1	-
50	1.439	1.018	1.426	1.008	-	-	-	112.9	102.7	-
58	1.439	1.018	1.424	1.007	-	-	-	108.2	54.9	-
60	1.439	1.018	1.418	1.003	-	-	-	-	64.9	-
65	1.439	1.018	1.419	1.003	-	-	-	-	49.3	-
70	1.220	1.018	-	-	1.414	1.000	-	-	45.8	-
75	-	-	-	-	1.414	1.000	-	-	-	58.6
80	-	-	-	-	1.415	1.000	-	-	-	63.4
85	-	-	-	-	-	-	-	-	-	90.4
90	-	-	-	-	-	-	-	-	-	117.1
95	-	-	-	-	-	-	-	-	-	160.0
100	-	-	-	-	-	-	-	-	-	161.9

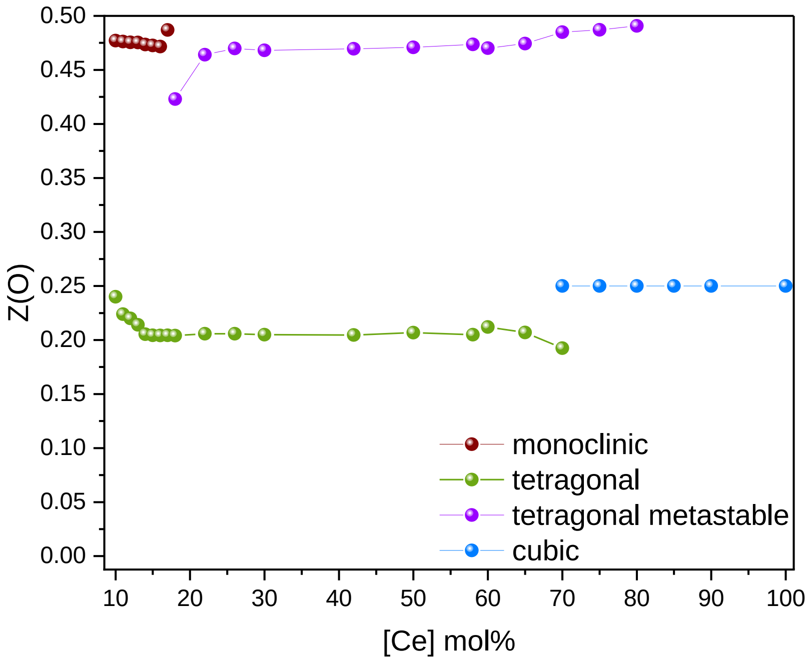

Figure S3: Z coordinates for the oxygen ions in $Zr_{1-x}Ce_xO_2$ ($0.1 \leq x \leq 1.0$).

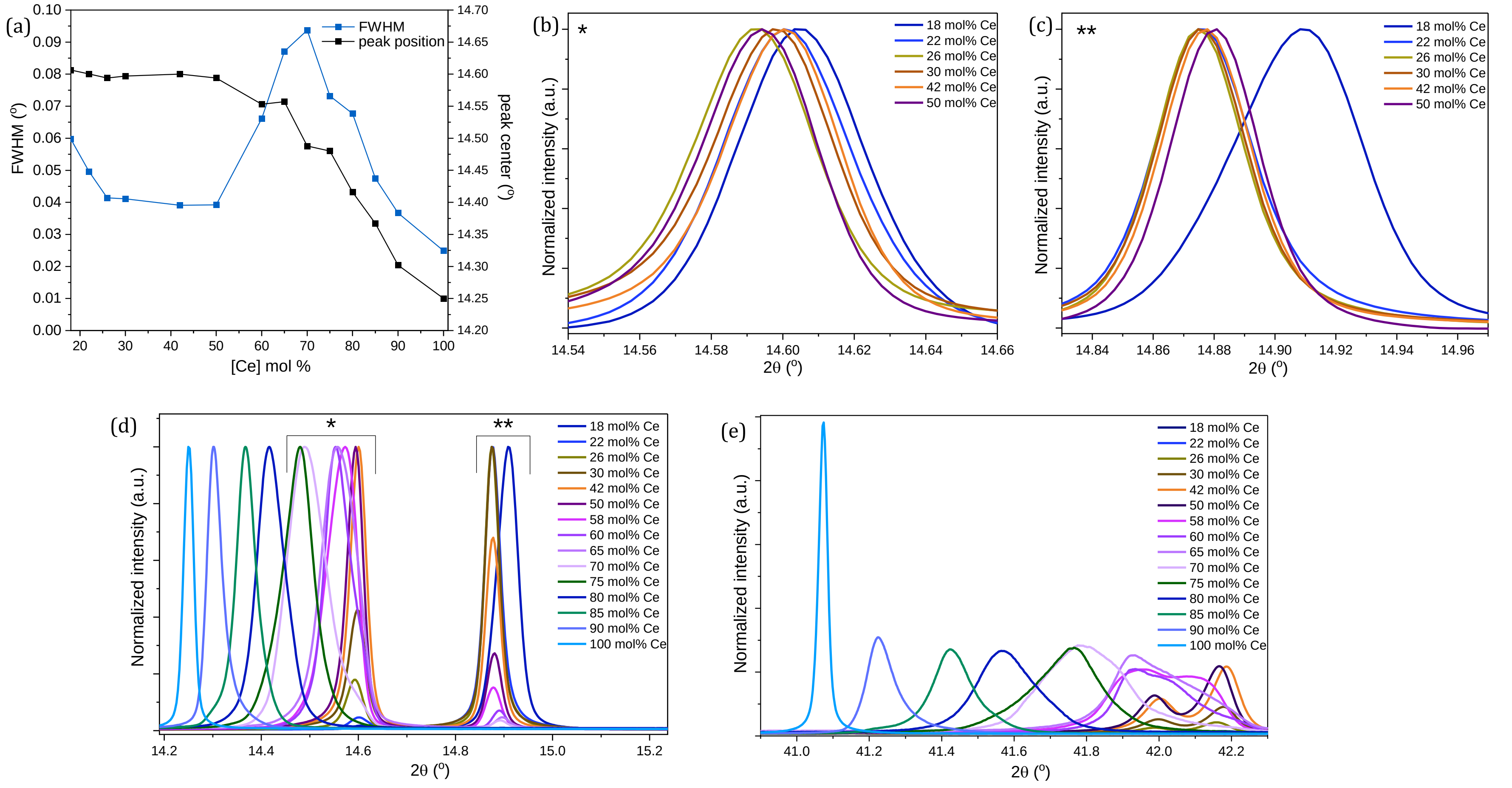

Figure S4: (a) FWHM and peak positions for the t', t'', and c peak in $Zr_{1-x}Ce_xO_2$ ($0.18 \leq x \leq 1.0$); (b) Normalized tetragonal metastable peak; (c) Normalized tetragonal peak of SPXRD diffractograms in $Zr_{1-x}Ce_xO_2$ ($0.18 \leq x \leq 0.50$); (d) Low 2θ and (e) High 2θ of SPXRD diffractograms in $Zr_{1-x}Ce_xO_2$ ($0.18 \leq x \leq 1.0$).

Raman and HERFD-XANES

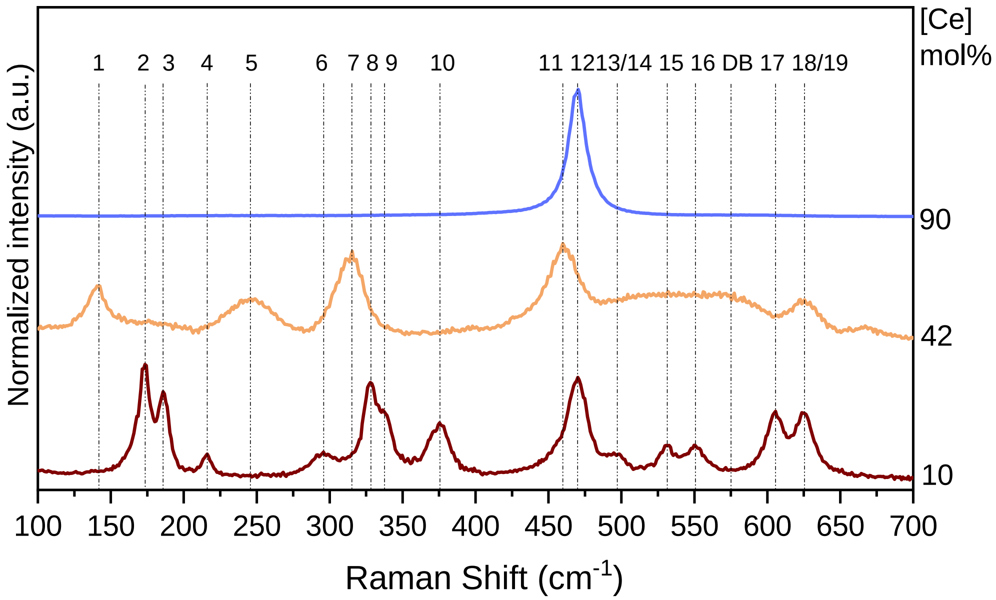

Figure S5: Raman spectra of 10, 42, and 90 mol% cerium-doped zirconia, with band assignment from the lowest to the highest frequency.

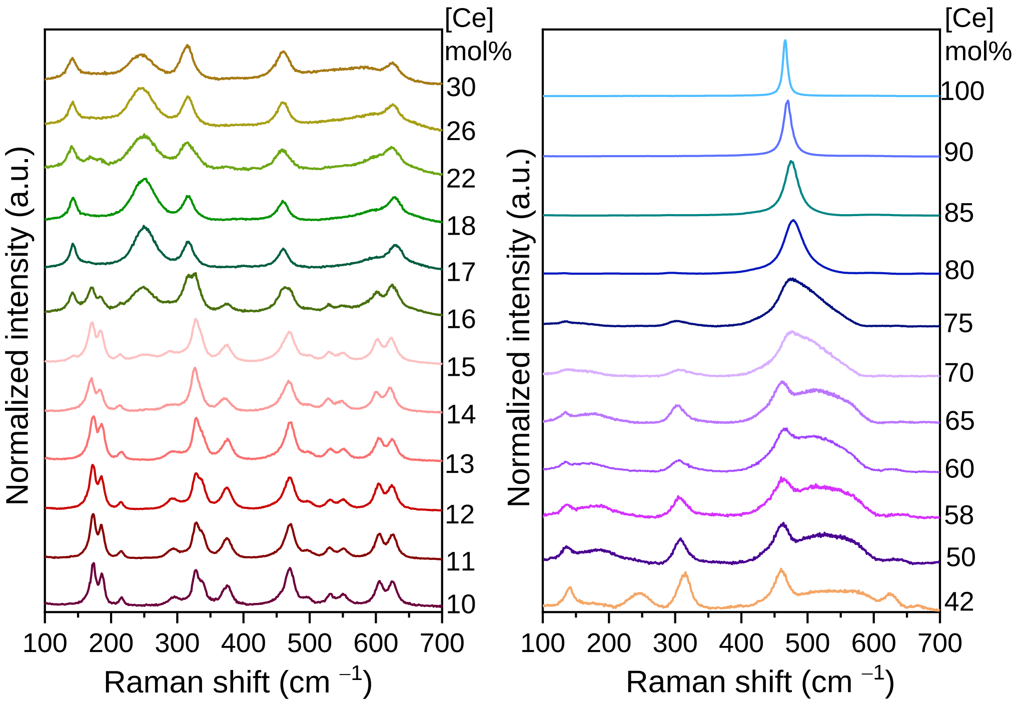

Figure S6: Raman spectra of all the synthesized $Zr_{1-x}Ce_xO_2$ ($0.1 \leq x \leq 1.0$) compositions.

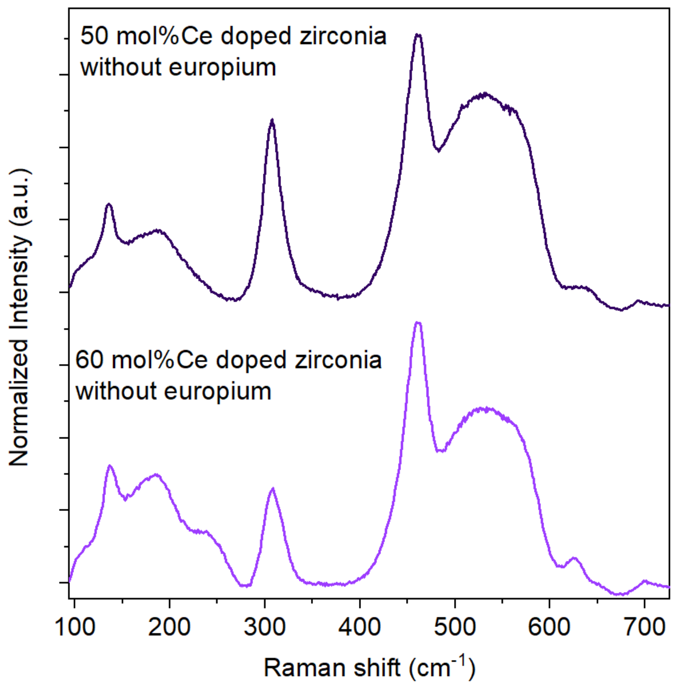

Figure S7: Raman spectra of 50 and 60 mol% Ce-doped zirconia without europium incorporation.

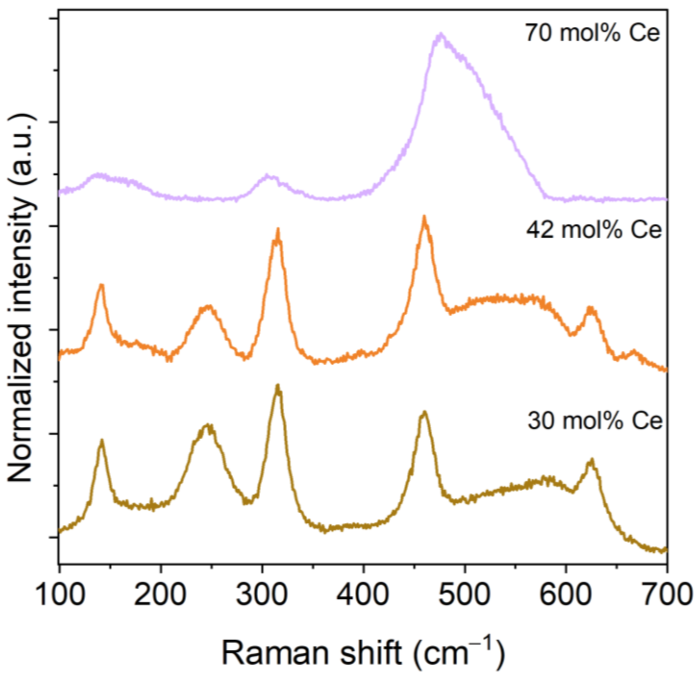

Figure S8: Raman spectra of 30, 42, and 70 mol% cerium-doped zirconia.

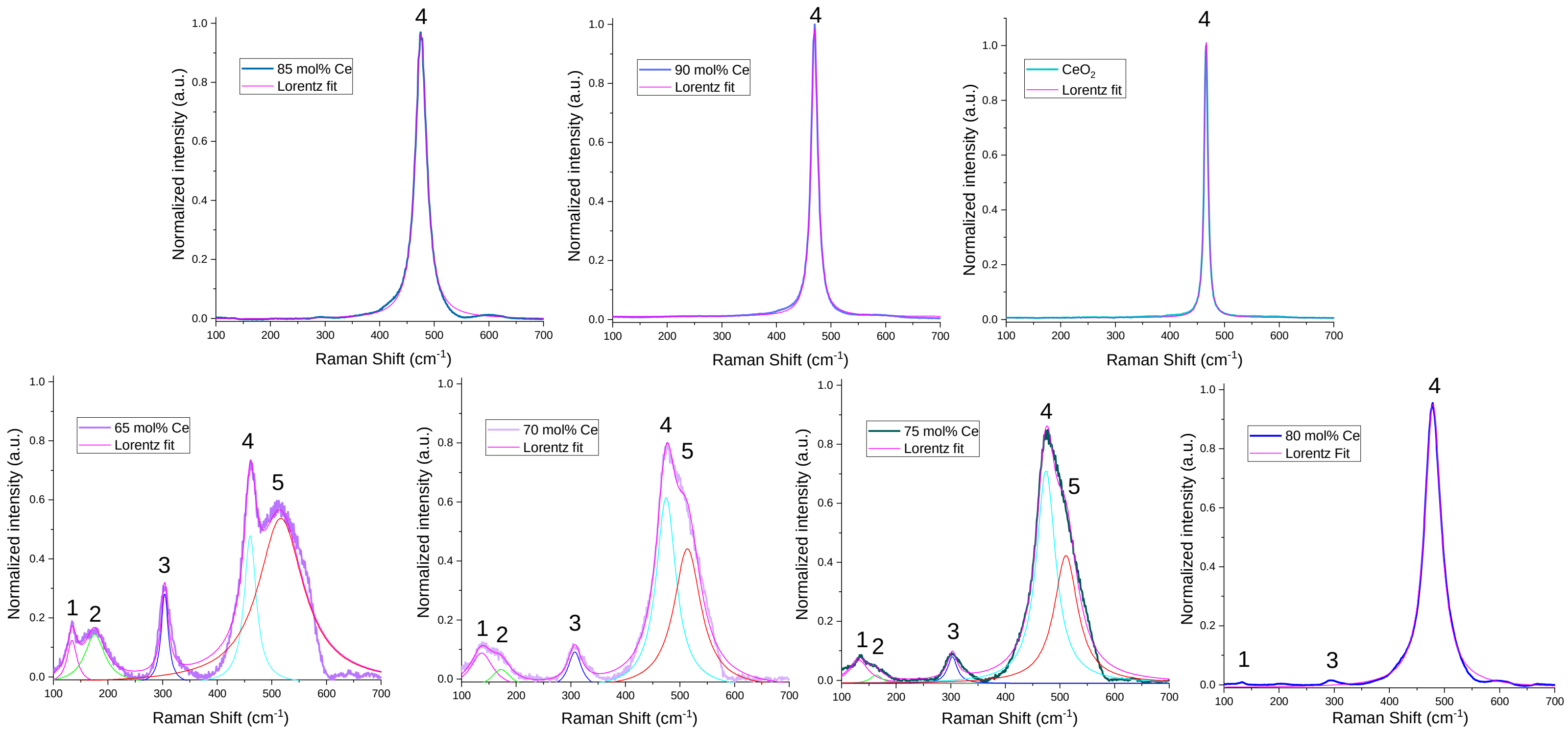

Figure S9: Lorentzian fitting of Raman peaks for the $Zr_{1-x}Ce_xO_2$ (0.65 ≤ x ≤ 1.0) compositions.

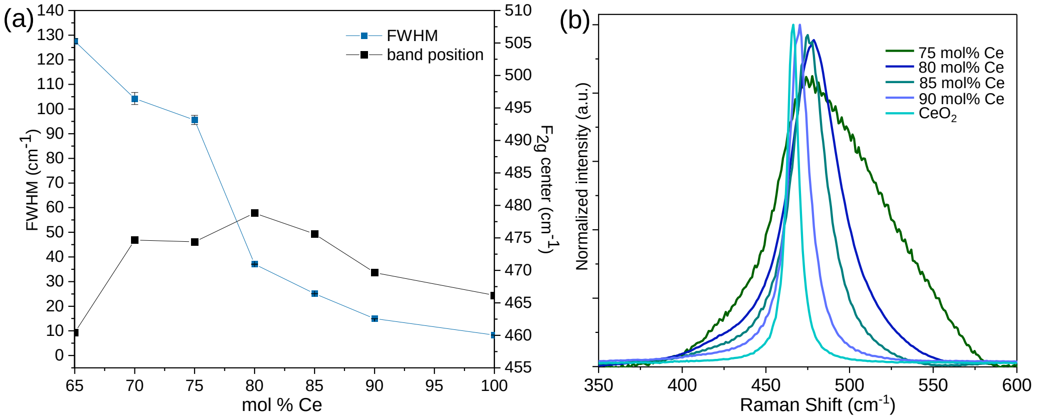

Figure S10: (a) FWHM and band position of the F_{2g} vibration mode in $Zr_{1-x}Ce_xO_2$ ($0.65 \leq x \leq 1.0$); (b) Raman spectra of $Zr_{1-x}Ce_xO_2$ ($0.75 \leq x \leq 1.0$).

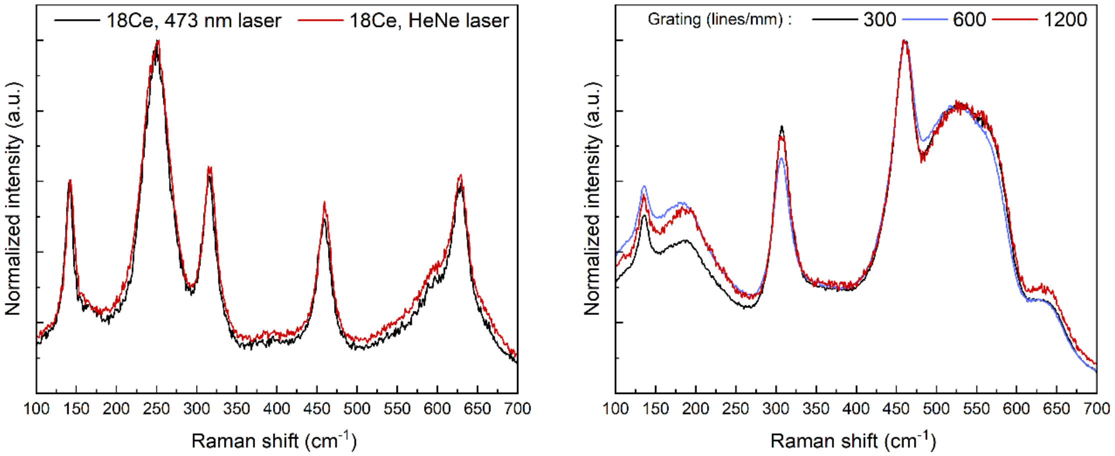

Figure S11: Raman spectra of the $Zr_{0.82}Ce_{0.18}O_2$ sample acquired using two different laser wavelengths (left), showing no significant variation in spectral features. Repeated measurements at the same sample spot using the HeNe laser under varying grating settings (right) demonstrate the stability of the spectra and indicate the absence of laser-induced modifications.

Luminescence Spectroscopy

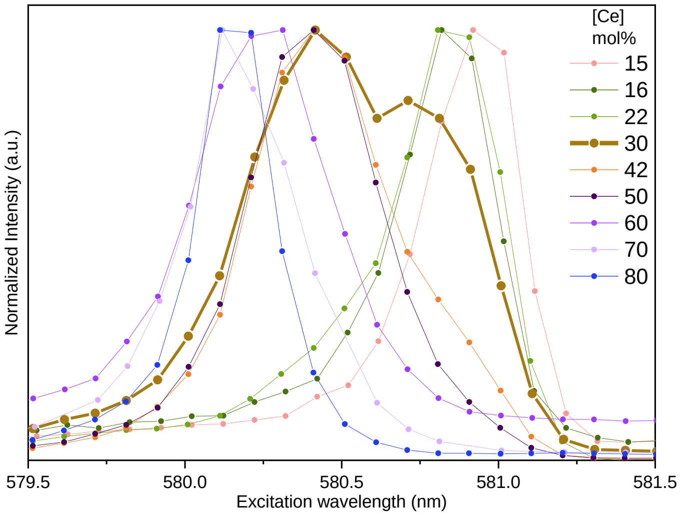

Figure S12: Excitation luminescence spectra of $Zr_{1-x}Ce_xO_2$ (x =, 0.15, 0.16, 0.22, 0.30, 0.42, 0.50, 0.60, 0.70, 0.75, 0.80, 0.90, and 1.0).

Decomposition of excitation spectra

The Lorentz mathematical model was used to decompose the excitation bands of $Zr_{1-x}Ce_xO_2$ (x = 0.15, 0.16, 0.22, 0.30, 0.42, 0.50, 0.60, 0.70, 0.75, 0.80, and 0.90) compositions. An example of the decomposition with emission spectra collected at different excitation wavelengths (numbered from 1 to 8) is presented in Figure S13. All decomposed spectra are shown in Figure S14 together with emission spectra collected at three different excitation wavelengths ($\lambda_{excitation}$). A broad low-intensity peak at around 580 nm (first peak in the excitation spectra (red traces)) is present in all compositions up to 90 mol% Ce, which can be associated with Eu^{3+} at the surface [5, 6]. The emission spectra related to this excitation wavelength show broader bands and the contributions from other emission bands, i.e. species present in the sample.

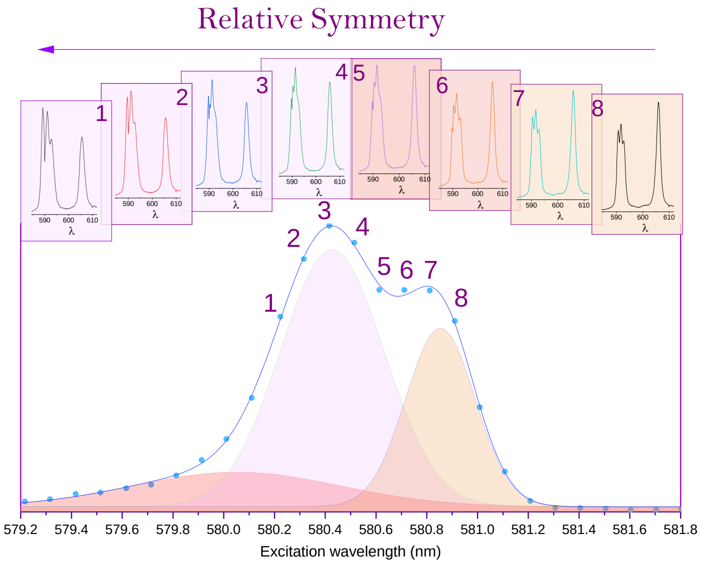

Figure S13: Excitation and respective luminescence emission spectra of Eu^{3+} in $Zr_{0.7}Ce_{0.3}O_2$.

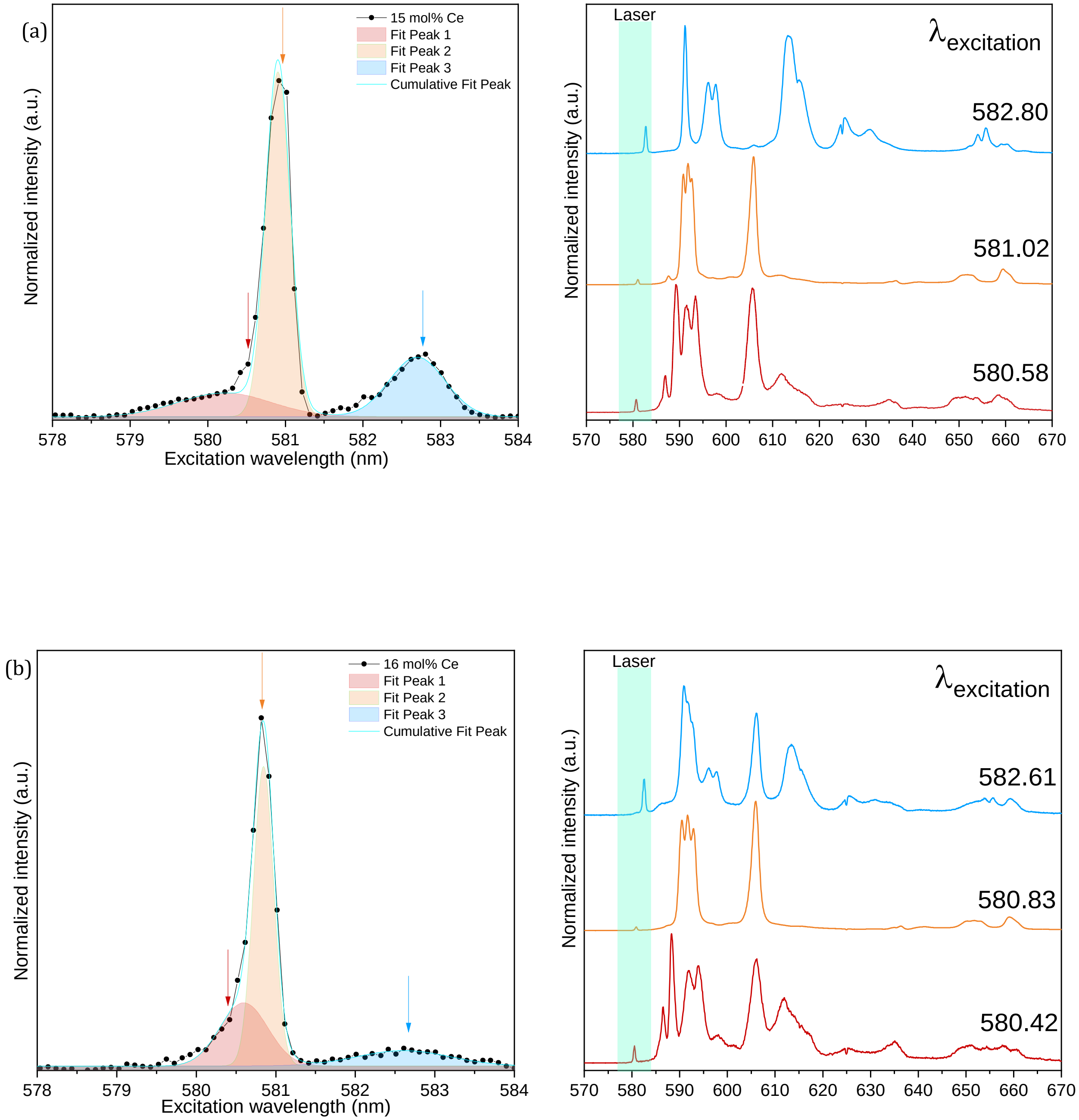

(a)
15 mol% Ce
Fit Peak 1
Fit Peak 2
Fit Peak 3
Cumulative Fit Peak
Normalized intensity (a.u.)
Excitation wavelength (nm)
Laser
λexcitation
582.80
581.02
580.58
(b)
16 mol% Ce
Fit Peak 1
Fit Peak 2
Fit Peak 3
Cumulative Fit Peak
Normalized intensity (a.u.)
Excitation wavelength (nm)
Laser
λexcitation
582.61
580.83
580.42

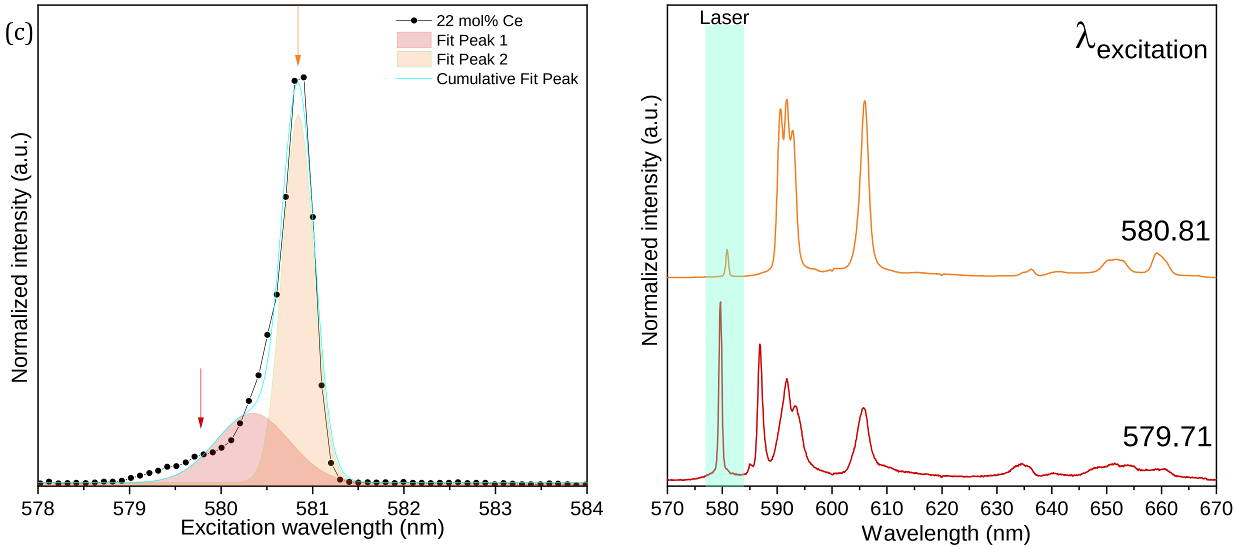
(c)
22 mol% Ce
Fit Peak 1
Fit Peak 2
Cumulative Fit Peak
Normalized intensity (a.u.)
578
579
580
581
582
583
584
Excitation wavelength (nm)
Laser
λexcitation
580.81
579.71
Normalized intensity (a.u.)
570
580
590
600
610
620
630
640
650
660
670
Wavelength (nm)

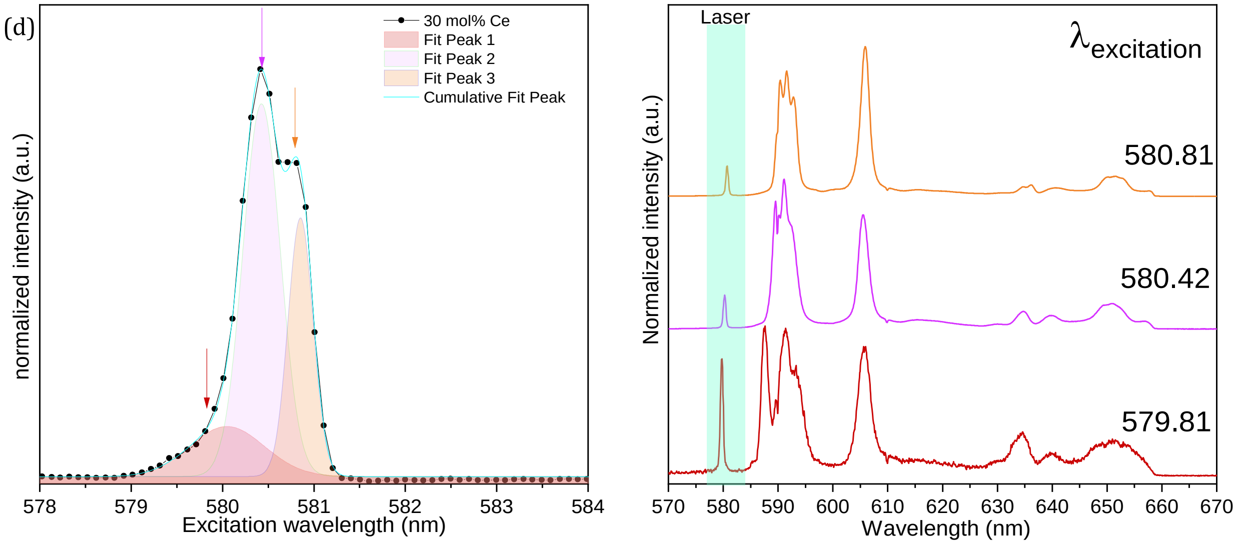
(d)
30 mol% Ce
Fit Peak 1
Fit Peak 2
Fit Peak 3
Cumulative Fit Peak
normalized intensity (a.u.)
578
579
580
581
582
583
584
Excitation wavelength (nm)
Laser
λexcitation
580.81
580.42
579.81
Normalized intensity (a.u.)
570
580
590
600
610
620
630
640
650
660
670
Wavelength (nm)

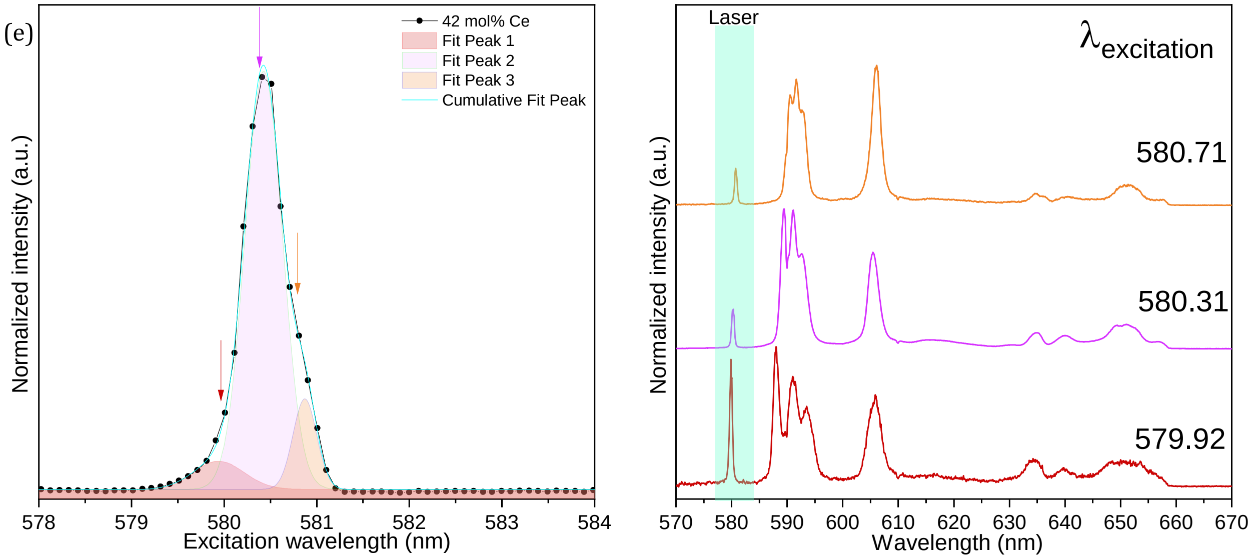
(e)
42 mol% Ce
Fit Peak 1
Fit Peak 2
Fit Peak 3
Cumulative Fit Peak
Normalized intensity (a.u.)
Excitation wavelength (nm)
578
579
580
581
582
583
584
Laser
λexcitation
580.71
580.31
579.92
Normalized intensity (a.u.)
Wavelength (nm)
570
580
590
600
610
620
630
640
650
660
670

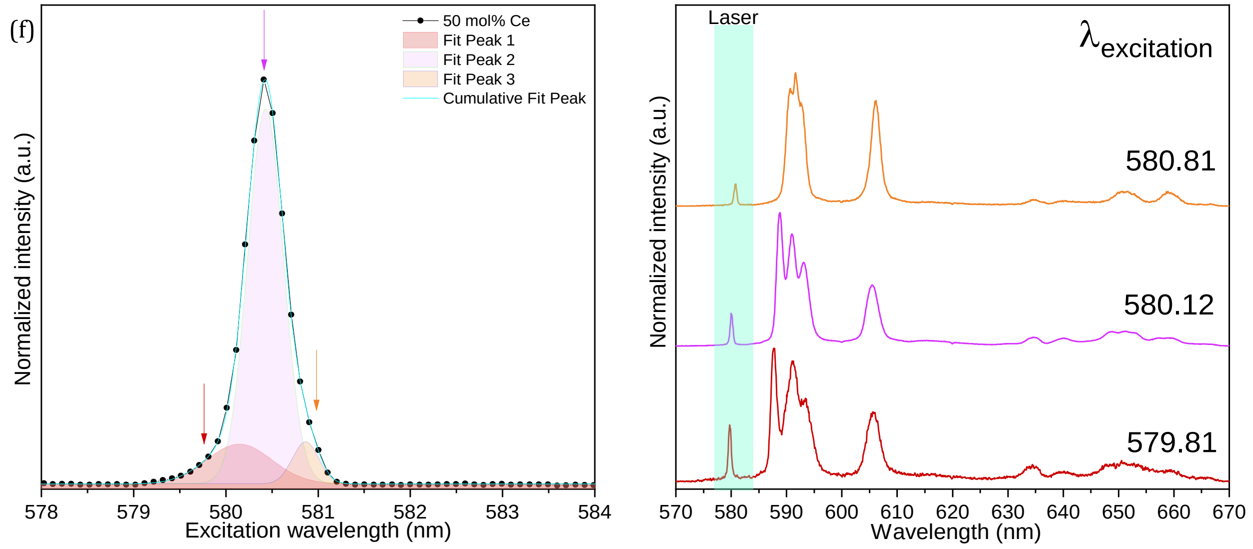
(f)
50 mol% Ce
Fit Peak 1
Fit Peak 2
Fit Peak 3
Cumulative Fit Peak
Normalized intensity (a.u.)
Excitation wavelength (nm)
578
579
580
581
582
583
584
Laser
λexcitation
580.81
580.12
579.81
Normalized intensity (a.u.)
Wavelength (nm)
570
580
590
600
610
620
630
640
650
660
670

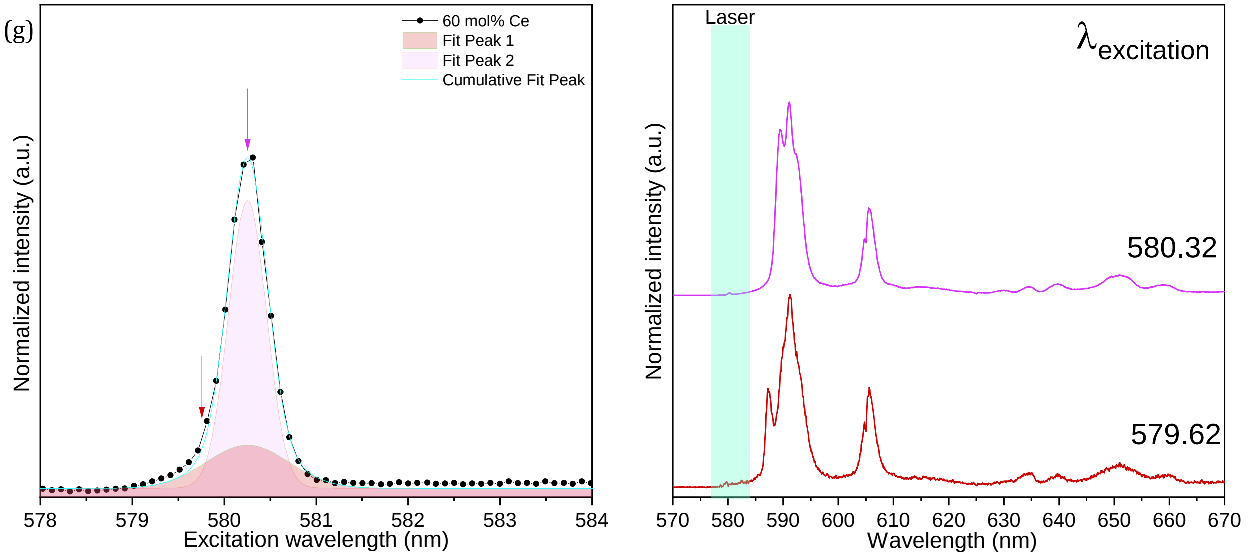
(g)
60 mol% Ce
Fit Peak 1
Fit Peak 2
Cumulative Fit Peak
Normalized intensity (a.u.)
Excitation wavelength (nm)
Laser
λexcitation
580.32
579.62
Wavelength (nm)

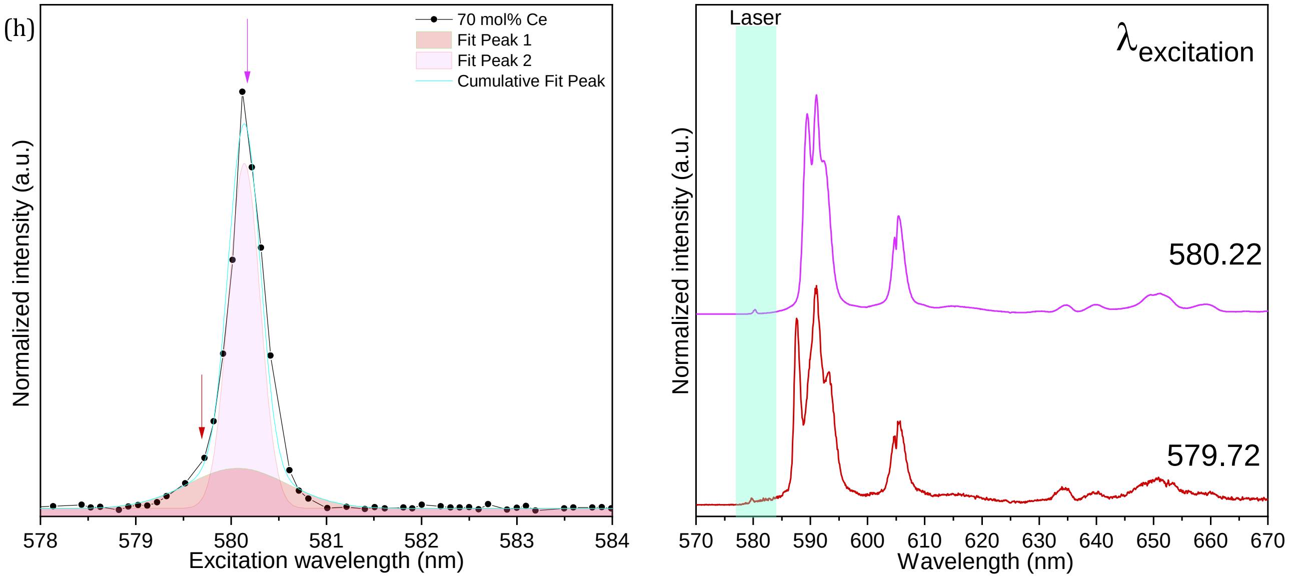
(h)
70 mol% Ce
Fit Peak 1
Fit Peak 2
Cumulative Fit Peak
Normalized intensity (a.u.)
Excitation wavelength (nm)
Laser
λexcitation
580.22
579.72
Wavelength (nm)

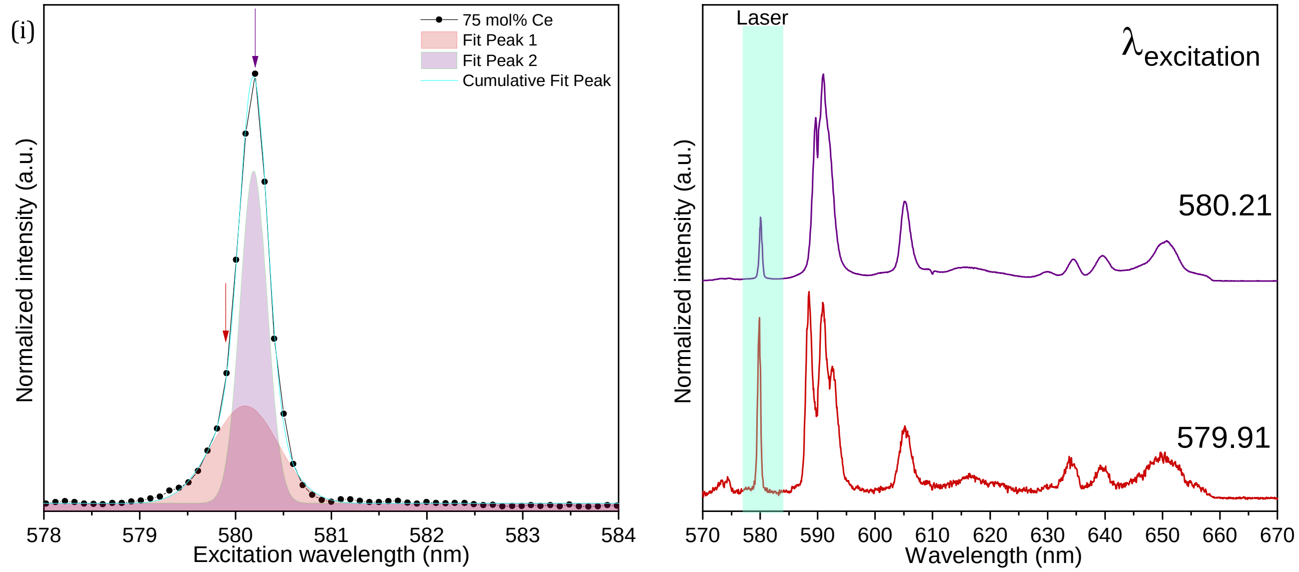
(i)
75 mol% Ce
Fit Peak 1
Fit Peak 2
Cumulative Fit Peak
Normalized intensity (a.u.)
578
579
580
581
582
583
584
Excitation wavelength (nm)
Laser
$\lambda_{excitation}$
580.21
579.91
570
580
590
600
610
620
630
640
650
660
670
Wavelength (nm)

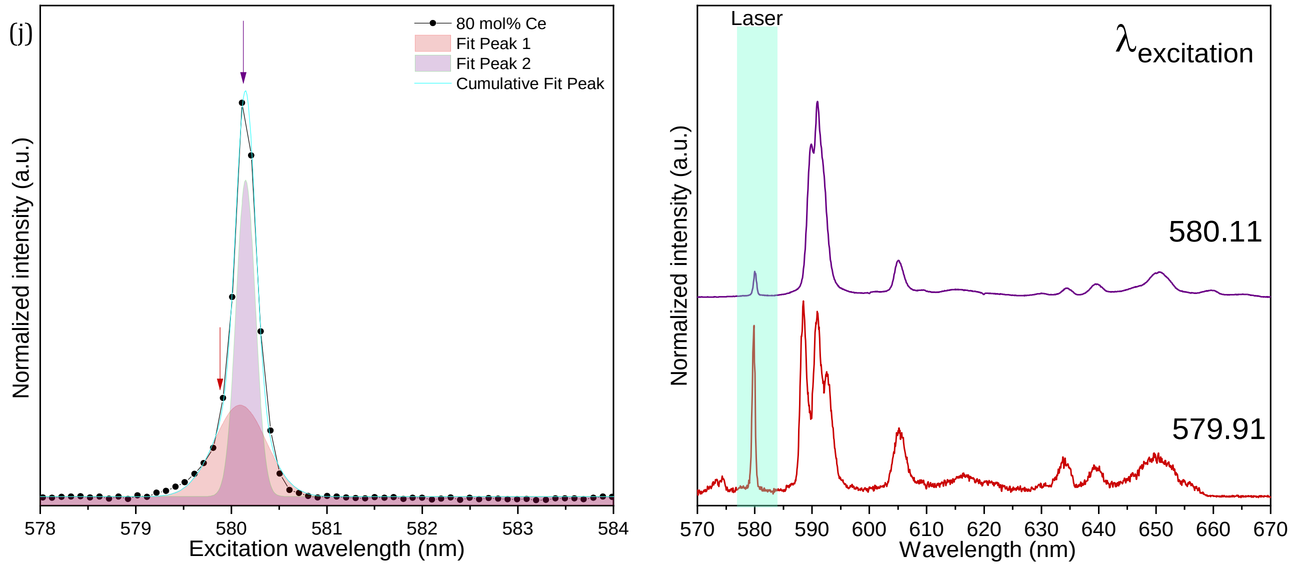
(j)
80 mol% Ce
Fit Peak 1
Fit Peak 2
Cumulative Fit Peak
Normalized intensity (a.u.)
578
579
580
581
582
583
584
Excitation wavelength (nm)
Laser
$\lambda_{excitation}$
580.11
579.91
570
580
590
600
610
620
630
640
650
660
670
Wavelength (nm)

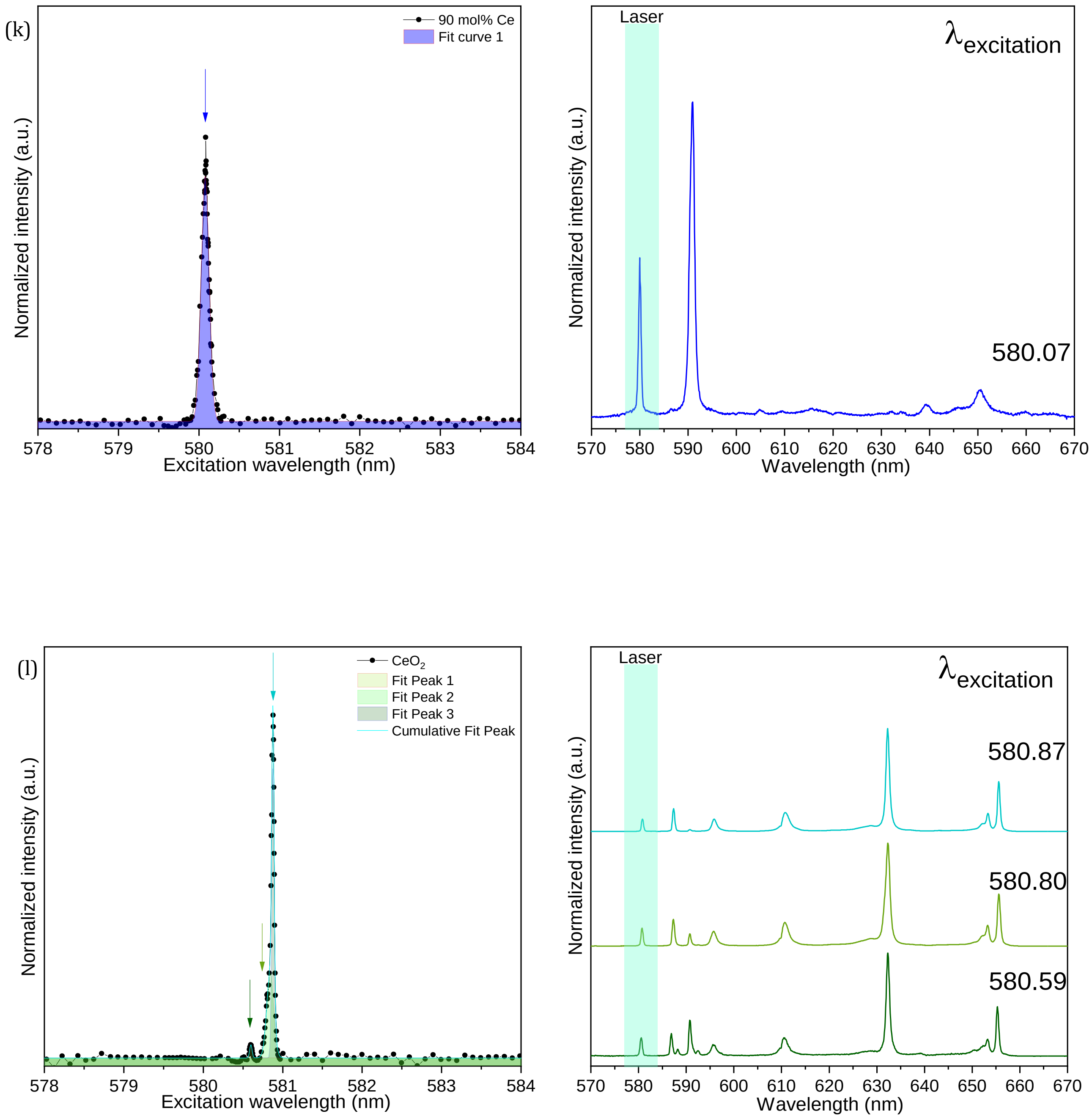

Figure S14: Excitation spectrum (left) and emission spectra collected at varying $\lambda_{excitation}$ (right) of synthesized $Zr_{1-x}Ce_xO_2$ (x = 0.15 (a), 0.16 (b), 0.22 (c), 0.30 (d), 0.42 (e), 0.50 (f), 0.60 (g), 0.70 (h), 0.75 (i), 0.80 (j), 0.90 (k), and 1.0(l)) compositions.

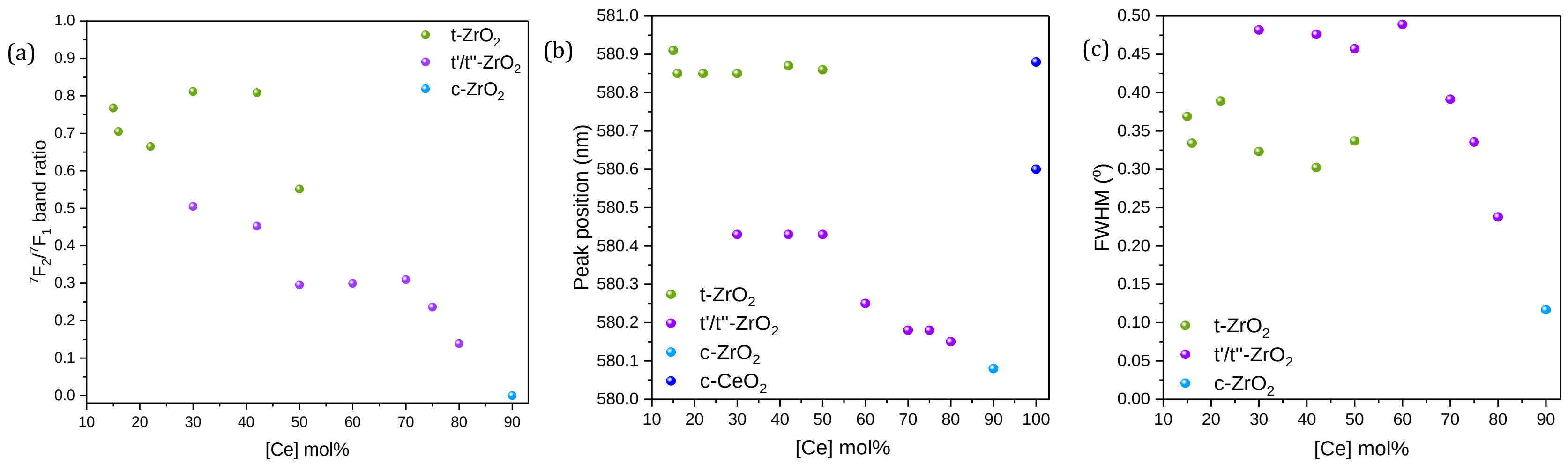

Figure S15: (a) Relative symmetry determined by the $^7F_2/^7F_1$ band ratio; (b) Peak position; and (c) FWHM of the excitation peak of $Zr_{1-x}Ce_xO_2$ (x = 0.15, 0.16, 0.22, 0.30, 0.42, 0.50, 0.60, 0.70, 0.75, 0.80, and 0.90).

References

1. Zhang, F.; Chen, C.-H.; Hanson, J. C.; Robinson, R. D.; Herman, I. P.; Chan, S.-W. Phases in Ceria–Zirconia Binary Oxide $(1-x)CeO_2$–$xZrO_2$ Nanoparticles: The Effect of Particle Size. *J. Am. Ceram. Soc.* **2006**, *89* (3), 1028-1036.

2. Yashima, M. Crystal Structures of the Tetragonal Ceria−Zirconia Solid Solutions $Ce_xZr_{1-x}O_2$ through First Principles Calculations $(0 \leq x \leq 1)$. *J. Phys. Chem. C* **2009**, *113* (29), 12658-12662.

3. Bogdan, M.; Peter, I. A Comprehensive Understanding of Thermal Barrier Coatings (TBCs): Applications, Materials, Coating Design and Failure Mechanisms. *Metals* **2024**, 14(5), 575.

4. Ida, T. Formula for the asymmetric diffraction peak profiles based on double Soller slit geometry. *Rev. Sci. Instrum.* **1998**, 69(6), 2268-2272.

5. Eibl, M.; Shaw, S.; Prieur, D.; Rossberg, A.; Wilding, M. C.; Hennig, C.; Morris, K.; Rothe, J.; Stumpf, T.; Huittinen. N. Understanding the local structure of Eu- and Y-stabilized zirconia: insights from luminescence and X-ray absorption spectroscopic investigations. *J. Mater. Sci.* **2020**, *55* (23), 10095-10120.

6. Montini, T.; Speghini, A.; Rogatis, L. D.; Lorenzut, B.; Bettinelli, M.; Graziani, M.; Fornasiero, P. Identification of the Structural Phases of $Ce_xZr_{1-x}O_2$ by Eu(III) Luminescence Studies. *J. Am. Chem. Soc.* **2009**, *131* (36), 13155-13160.
